# Niche Shifts Induce Major Changes in the Ranges of the World's Worst Invasive Ant Species

**DOI:** 10.1002/ece3.71754

**Published:** 2025-07-08

**Authors:** Qiance Wei, Xueyou Zhang, Xiaokang Hu, Jianmeng Feng

**Affiliations:** ^1^ College of Agriculture and Biological Science Dali University Dali Yunnan China; ^2^ Research Center for Agroecology in Erhai Lake Watershed Dali University Dali China; ^3^ Cangshan Forest Ecosystem Observation and Research Station of Yunnan Province Dali University Dali Yunnan China

**Keywords:** amplification effects, climatic niches, climatic ranges, global dynamics, invasive ants

## Abstract

Invasive ants have exerted major effects on global ecosystems and economic systems. Therefore, their future niche and range shifts have received more research attention; however, the shifts between their native and introduced populations have not been widely investigated. Here, we examined niche and range shifts between native and introduced populations of 18 IUCN‐recognized invasive ant species based on 133,786 global occurrence records. Most introduced populations have undergone substantial niche and range expansions compared to their native counterparts. *Tapinoma melanocephalum* , *Paratrechina longicornis* , *Lasius neglectus*, and *Acromyrmex octospinosus* had the largest introduced potential ranges, expanding ranges, and centroid shifts in their niches and ranges, respectively, suggesting that their invasion risk is high and thus that they require increased attention. Introduced range overlap was greatest in the southeastern USA and Europe, and the largest areas of the expanding range were observed in the southeastern USA, Mexico, and Brazil, indicating that these are the priority regions for combatting their impacts. Additionally, we detected strong positive associations between their niche and range shifts, and small niche shifts induced large range shifts.

## Graphical abstract

We detected strong positive associations between their niche and range shifts. Small niche shifts induced large range shifts in them.

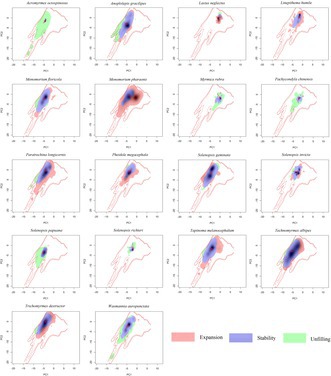

## 1 Introduction

Invasive ants (Hymenoptera, Formicidae) have posed significant threats to global ecosystems and economic systems due to their high invasiveness and deleterious effects on native ant populations (Lach and Hooper‐Bui 2010; Gruber et al. 2021; Angulo et al. 2022; Xu et al. 2022). Their invasions often result in the decline of native species and alterations in ecosystem services. For example, previous studies have shown that *Solenopsis invicta* outcompetes native ants in various habitats, which can lead to the collapse of food webs and biodiversity loss (Turner et al. 2021; Vinson 2021; Lin 2023). In agricultural systems, invasive ants have been linked to crop damage, as they protect pest species (e.g., aphids) from predators, which promotes increases in pest populations (Anjos et al. 2021; Lach 2021; Gruber et al. 2022). Additionally, invasive ants can alter the composition of soil and nutrient cycling, which can further impact agriculture and biodiversity (Siddiqui et al. 2021; Lee and Yang 2022; Shi et al. 2023; Tercel et al. 2023; Wong et al. 2023). There is thus a need to assess the invasion risk of invasive ants.

Climatic conditions have major effects on the life history, including the distribution, reproduction, and even survival of ants (Menzel and Feldmeyer 2021; Nascimento et al. 2022; Parr and Bishop 2022; Menchetti et al. 2024). Therefore, their invasion success or invasion risk, to a great extent, could be determined by their adaptability to climatic conditions (Bates et al. 2020; Lach 2021). Many studies have highlighted the role of climatic conditions in ant invasions (e.g., Chen 2008; Bertelsmeier et al. 2015; Buczkowski and Bertelsmeier 2017; Sung et al. 2018; Cordonnier et al. 2020; Lach 2021; Jung et al. 2022; Merchlinsky et al. 2023; Li et al. 2024). For instance, Song et al. (2021) found that future climate changes could induce the range expansions of *Solenopsis invicta* in the south and southeast of China, and Lee et al. (2022) projected that under future climate change scenarios, *Solenopsis invicta* would expanded its ranges, whereas for *Anoplolepis gracilipes* its ranges might remain stable. Therefore, the roles of climatic adaptation in invasion risk assessments should not be underestimated, and further investigation is needed.

Given that the climatic niche and range dynamics generally reflect shifts in their climatic adaptability, they have generally been used to evaluate their invasion risks under changing climatic conditions. Therefore, many studies have characterized their niche and range dynamics and the roles of climate change on them (e.g., Song et al. 2021; Li et al. 2023, 2024). For example, Nair et al. (2024) detected substantial future warming‐induced range expansions in Argentine ants ( *Linepithema humile* ), and Li et al. (2023) identified substantial niche and range shifts in *Solenopsis invicta* in China, suggesting that both *Linepithema humile* and *Solenopsis invicta* have high invasion risk in the future. The identification of niche shifts between the native and introduced/invasive ants could offer important information for assessing their invasion risk (Bates et al. 2020), and therefore this topic has received much attention in the past. For example, Kumar et al. (2015) detected substantial niche and range shifts between native and introduced populations of *Nylanderia fulva*, and Bujan et al. (2021) identified thermal niche expansions of invasive populations of *Tapinoma magnum* compared to its native populations. Additionally, Bates et al. (2020) compared the niche shifts between native and invasive ants and found that invasive ants experienced smaller climatic niche shifts than non‐invasive alien ant species. However, until now few studies have investigated the associations between niche and range shifts between introduced and native populations of invasive ants.

Recently, how niche shifts in invasive species influence their ability to establish and expand ranges in new environments has received increased attention (e.g., Petitpierre et al. 2012; Liu et al. 2020; Lo Parrino et al. 2023). Ecological niche modeling (ENM) is often used to predict species' potential ranges under the hypothesis of niche conservatism, which suggests that a species' ecological niche is relatively unchanged across time and space (Guisan et al. 2014; Kolanowska and Konowalik 2014; Liu et al. 2020). This hypothesis underlies the use of ENMs to forecast how climate change might alter invasive species' potential ranges by inducing shifts in their climatic niches while maintaining their ancestral preferences (Sexton et al. 2017; Jourdan et al. 2021; Nie and Feng 2022). However, the validity of the hypothesis of niche conservatism has sparked considerable debates (Parravicini et al. 2015). Although some studies support the idea that niches remain stable (Atwater et al. 2018; Montagnani et al. 2022; Nie and Feng 2022), others provide evidence to the contrary, showing that niches can change significantly over time or across environments (Beukema et al. 2018; Lörch et al. 2021; Cao et al. 2022). This controversy raises concerns about the reliability of ENMs for accurately calibrating the range shifts in invasive species. Moreover, trends in niche conservatism may vary with the biome (Liu et al. 2020), which underscores the importance of conducting biome‐specific investigations. Although niche conservatism has received much research attention, few studies have probed the range conservatism of invasive species, especially that of invasive ants.

Given the close associations between niche and range shifts in invasive species, some researchers have proposed that niche and range shifts could be used to assess the risks associated with invasive species (e.g., Kolanowska and Konowalik 2014; Jourdan et al. 2021; Malone et al. 2023; Li et al. 2024). Even small shifts in the ecological niches of invasive species can lead to large range shifts. For example, Cao et al. (2022) found that small climatic niche shifts of *Eucalyptus globulus* (Tasmanian blue gum), an invasive tree, could induce large range shifts between the introduced populations and its native counterparts, which, to a certain extent, were echoed by a study on the niche shifts in invasive giant African snails (*Lissachatina fulica*) (Wu et al. 2023). However, to our knowledge, few studies have investigated the niche and range dynamics of the world's worst invasive ants relative to their native counterparts.

Here, we developed a unified scheme (unified data sources, candidate predictors and range dynamic models) to compare the niche and range dynamics of 18 major invasive ants recognized by the International Union for Conservation of Nature (IUCN) and their native counterparts and tested whether small niche shifts could induce large range shifts in most of the world's worst invasive ants, as well as test their niche and range conservatism. We hope our study could deepen our understanding of the mechanisms responsible for the niche and range shifts in the invasive ants relative to their native populations.

## 2 Materials and Methods

### 2.1 Occurrence Data of the Worst Invasive Ants

**FIGURE 1:**
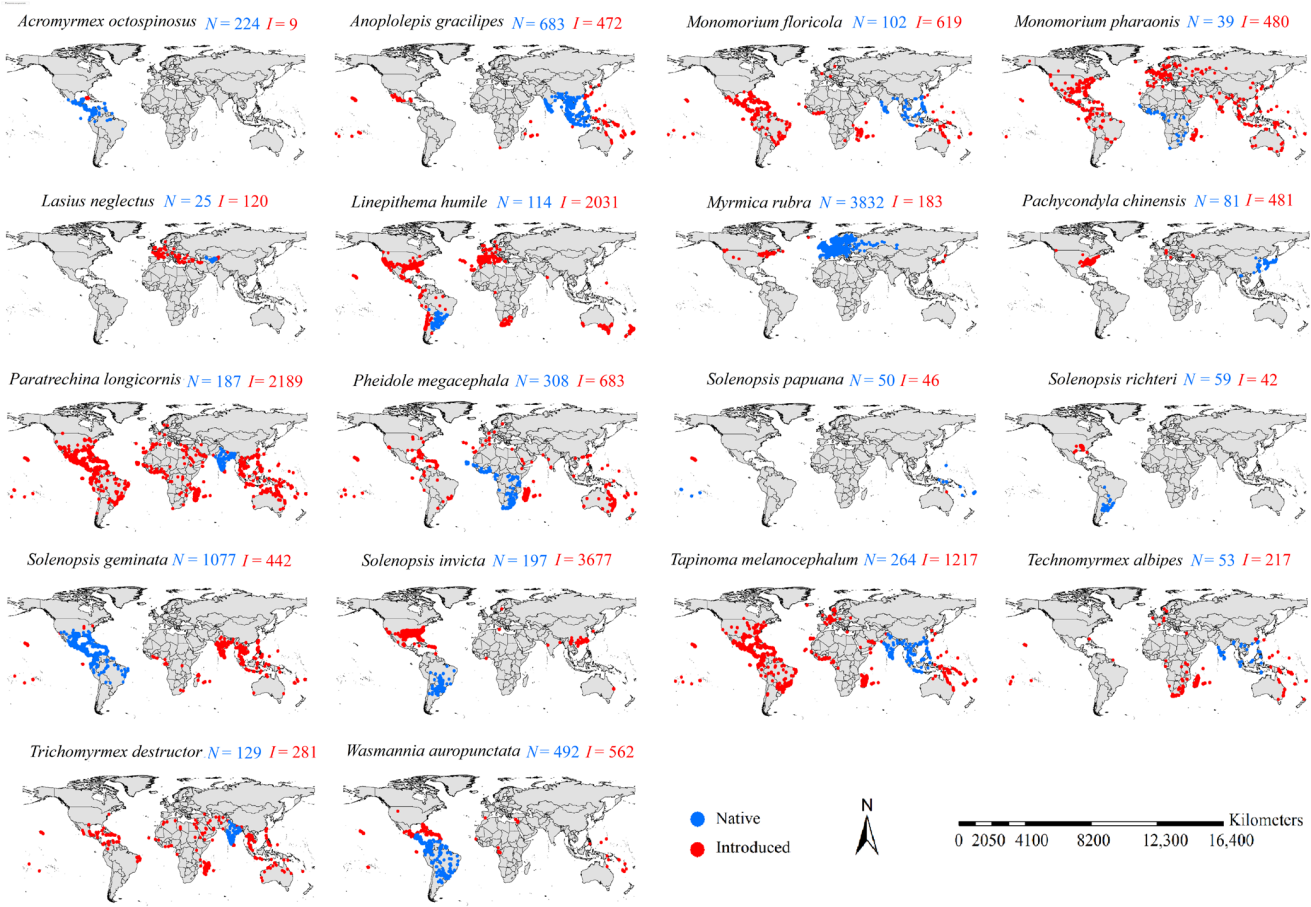
Occurrence maps of the 18 major ant invasive species. The occurrences were obtained through extensive survey in literature and online datasets. Totally, we retrieved 133,786 occurrence points which were reduced to 21,710 after spatial rarefaction. Blue and red point indicated the native and introduced occurrences, repsectively, and blue *N* and red *I* represented the amounts of native and introduced occurrences, repsectively.

From the Global Invasive Species Database (GISD) sponsored by the IUCN (https://www.iucngisd.org/gisd/, accessed on August 1st, 2024), we obtained a list of the 19 world's worst invasive ant species that have caused substantial ecological and economic damage (Table S1). Next, from multiple sources, including a comprehensive review of published literature and accessible online databases (Table S2), we compiled a vast array of 133,786 occurrence records of invasive ant species (DOI: 10.6084/m9.figshare.29191778). For each species, we constructed an initial dataset of occurrences. Following the methodological framework proposed by Zhou et al. (2024), we ensured the accuracy of the data by retaining only records with a geographic‐coordinate‐uncertainty of > 5 km. Species observations and occurrence recording tend, inevitably, to be clustered around field camps, observation routes and settlements (Pearson et al. 2007). Therefore, sample bias, to a great extent, could be characterized by occurrence cluster, resulting in the possibility of inflating validation statistics caused by including localities that are spatially dependent (Hampe 2004; Luoto et al. 2005). Hence, spatial rarefaction could be one of the major methodologies to reduce the effects of sample bias (Brown et al. 2017; Kotlov and Chernenkova 2020). Through SDM toolbox, a python‐based GIS toolkit for species distribution modes (Brown et al. 2017), we used a 5 km radius to spatial rarefy occurences of each species, retaining only one record in each grid cell of 5 × 5 km (Pearson et al. 2007; Zhang et al. 2024). We removed the Caribbean crazy ant (*Nylanderia pubens*) from our formal analyses because of its occurrence record scarcity (> 30 records after the spatial rarefication). This resulted in the identification of 18 major invasive ants (Figure 1). Finally, these species had 21,710 after spatial rarefaction. Finally, for each of these 18 species, we developed a refined and high‐quality occurrence dataset, which provided a robust foundation for subsequent niche and range dynamic analyses. Then, with a reference to the GISD' delimitations of each species' introduced and native regions, we divided the occurrence dataset into native and introduced occurrences for each species (Figure 1).

### 2.2 Climatic Predictors in the Models

To ensure consistency with the temporal distribution of species occurrence records—over 90% of which were documented after 1990—we selected climatic datasets from 1991 to 2020. These datasets, sourced from the Climatic Research Unit (CRU), were processed to generate average values for 19 bioclimatic predictor variables. The data were formatted into grid layers with a spatial resolution of approximately 10 km (5 arc‐minutes). The 19 bioclimatic variables are consistent with those from WorldClim 2.1 (Fick and Hijmans 2017) and encompass a wide range of climatic factors, including 11 related to energy availability and 8 associated with precipitation. These variables capture various aspects of climate: annual averages (e.g., mean annual temperature, total annual precipitation), seasonal variations (e.g., temperature seasonality, precipitation seasonality), quarterly means (e.g., averaged temperature in the driest seasons, averaged temperature in the warmest seasons), and extreme values (e.g., maximum temperature in the hottest months, minimum temperature in the coldest months). This selection of climatic predictors provides a comprehensive representation of global climate conditions, which helps ensure the ENMs' reliability developed in this study.

### 2.3 Addressing Multicollinearity Among the Predictors in ENMs

To minimize the effects of predictor multicollinearity on the ENMs used to investigate the range shifts in each species, we employed a methodology recommended by Gong et al. (2020). First, for each invasive ant, we constructed preliminary ENMs using native occurrence records to assess the relative importance of 19 bioclimatic predictors in Biomod2 (Thuiller et al. 2009), and variable importance values (IVs) were calibrated through five permutation iterations (Table S3). Next, to mitigate the impacts of collinearity among predictors, we applied a filtering process based on guidelines from Dormann et al. (2013), in which variables in pairs with correlation coefficients of ≥ 0.7 and lower IVs were removed (Tables S3 and S4). Of note, in this process, Pearson correlation coefficients were adopted when the variables obeyed the normal distribution, or Spearman correlation coefficients were used. This threshold ensures that ENMs retain predictive accuracy and avoids excessive redundancy among predictors. Dormann et al. (2013) highlighted that this approach balanced model performance with the need to reduce multicollinearity and outperformed other specialized collinearity‐reduction techniques. The remaining variables were then incorporated into ENMs to delineate the potential distribution of the invasive ant in its native range. The same approach was applied when selecting predictors for modeling the range of introduced populations.

### 2.4 Predicting the Potential Ranges

Machine learning methods have recently demonstrated exceptional performance in various applications and are increasingly used for ecological modeling (Kouadri et al. 2021; Hai et al. 2022). To address variability among individual modeling techniques and identify consistent patterns (Shamshirband et al. 2019), the present study utilized the Biomod2 platform, an advanced tool for ecological niche modeling (ENM) (Thuiller et al. 2009). For native or introduced populations of each invasive ant, Biomod2 was applied to predict the global climatic range of each invasive ant. We adopted an ensemble modeling approach to improve prediction reliability and minimize uncertainty (Oppel et al. 2012; Shabani et al. 2016). Our ensemble ENMs incorporated 10 algorithms (Table S5) to ensure robust and reliable results. We followed the methodology of Zhou et al. (2024) to generate pseudo‐absence records (PAs). For species with more than 1000 occurrence records, an equal amount of PAs was selected randomly five times. When the amount of occurrences was fewer than 1000, we generated 1000 randomly selected PAs in five iterations. Equal weightings were applied to both presence and pseudo‐absence records to balance the dataset (Nie et al. 2024). To evaluate the reliability of the ENMs, we used cross‐validation techniques to measure the divergence of model predictions from random expectations. We adopted a five‐fold cross‐validation method to assess model reliability. In this method, the occurrence dataset of each species was equally divided into five folds. Each fold is used once as the validation (test) set, whereas the remaining four folds are used for model calibration (training). This process is repeated five times, ensuring each fold serves as the validation set exactly once. Finally, we averaged the five validation results. This method assesses model performance, reduces overfitting, and evaluates transferability in geographic or environmental space, providing a robust estimate of model accuracy on unseen data (Thuiller et al. 2009; Gong et al. 2020). Model reliability was calibrated by two metrics: AUC and TSS. These metrics are widely used for evaluating ENM performance and are complementary; they thus serve as cross‐validation tools (Shabani et al. 2018). We excluded algorithms that did not meet the thresholds proposed by Cao et al. (2022), specifically AUC < 0.8 and TSS < 0.6 (Table S6). Of note, AUC has some weaknesses, such as insensitivity to probability calibration and fit quality, emphasis on ROC space regions of limited ecological relevance, equal weighting of omission and commission errors, and strong dependence on the spatial extent of absence or background data (Lobo et al. 2008). Therefore, weights proportional to each model's TSS were assigned to produce an ensemble projection instead of AUC.

Additionally, the maximization‐sensitivity‐specificity‐sum threshold (MSS threshold), which is known for its robustness across most data types (Liu et al. 2016), was applied to determine the ranges of native and introduced populations of each species.

### 2.5 Evaluating Niche Dynamics

There are three primary methods for analyzing niche dynamics: the ordination approach, the univariate approach, and the ENM approach. Among these, the COUE framework is widely recognized as the benchmark methodology, given its reliability, user‐friendly implementation, and effectiveness in mitigating sampling bias and resolution‐related discrepancies (Guisan et al. 2014). Using the COUE framework in the R package, that is, Ecospat developed by Di Cola et al. (2017), we investigated the niche dynamics of the invasive ant species relative to its native counterparts. Specifically, two PCA axes were generated to define the species' niche, and the contributions of the predictors to the PCA were analyzed. As suggested by the COUE scheme (Broennimann et al. 2012; Guisan et al. 2014), we partitioned a species' niche into three elements: expansion (E), unfilling (U), and stability (S). The element U represents portions of the niche only occupied by the native population, E includes portions of the niche exclusive to the introduced population, and S refers to the niche portions shared by both native and introduced populations. The combined total of S and E represents the overall niche space of the introduced populations, whereas the sum of U and S defines the native niche space. To measure changes in niche breadth, we calculated the niche breadth ratio (*NBR*):

$$\mathrm{NBR}=\frac{\mathrm{NBI}}{\mathrm{NBN}}$$

where *NBI* and *NBN* represent the niche breadths of the introduced and native populations, respectively.

To assess shifts in niche positions, we applied the niche similarity index (*NSI*):

$$\mathrm{NSI}=\frac{2\mathrm{NBS}}{\mathrm{NBI}+\mathrm{NBN}}$$

where *NBS* represents the niches shared by the introduced and native populations. If *NSI* > 0.5, the introduced and native populations have similar niche positions.

If *NSI* < 0.5 and *NBR* > 1, the results would not support the hypothesis of niche conservatism (Broennimann et al. 2007). Conversely, the hypothesis of niche conservatism would be confirmed if the introduced population's niche breadth is narrower than that of the native population, or when the two populations occupy similar niche positions.

### 2.6 Evaluating Range Dynamics

In the present study, we created range ratio index (*RRI*) and range similarity index (*RSI*) to calibrate the range expansions of the invasive ant species and the position shifts between the native and introduced populations of each species, respectively. Firstly, we calculated the area of native and introduced ranges and the area of ranges shared by the native and introduced populations of each species, represented by *RN*, *RI*, and *RS*, respectively. Then, we used *RN* and *RI* to calculate *RRI* through the formula:

$$\mathrm{RRI}=\frac{\mathrm{RI}}{\mathrm{RN}}$$

We used *RN*, *RI* and *RS* to calculate *RSI* as follows:

$$\mathrm{RSI}=\frac{2\mathrm{RS}}{\mathrm{RI}+\mathrm{RN}}$$

If *RSI* equals 1, no position shifts occur between native and introduced ants, while *RSI* equals 0, complete position shifts occur. Additionally, if *RSI* < 0.5, the native and introduced ranges were in different positions.

If *RRI* < 1 or *RSI* > 0.5, this suggests that the introduced population's range is largely conserved from the native range, supporting the hypothesis of range conservatism.

Additionally, we also overlapped native range with introduced one for each species, separately. Through this method, we retrieved its expanding range which was solely occupied by its introduced population, and estimated its area. Then, we overlapped expanding ranges of all 18 species.

### 2.7 Effects of Predictor Ranges on the Range and Niche Shifts

From the PCA analysis in COUE scheme, we determined the top predictor underlying the niche shifts of each species. For each species, we then extracted min and max values of the top predictor of the introduced and native occurrences and calculated the ranges of the top predictors of the native and introduced occurrence records separately. We calculated ratios of the top predictor ranges (the introduced ranges divided by the native ones) for each species. We then used correlation analysis to examine associations of the top predictor range ratios with niche breadth ratios and range ratios. We also conducted correlation analyses to characterize associations between niche breadth ratios and range ratios and between niche similarity indices and range similarity indices. Of note, in these analyses, Spearman correlation coefficients were adopted when the variables did not obey the normal distribution, or Pearson correlation coefficients were used.

## 3 Results

### 3.1 Model Reliability

The portions of the two most significant PC axes responsible for the niche dynamics between the major invasive ant species in native and introduced regions ranged from 57.5% to 77.9%, with an average of 68.43% ± 5.51% (Table S7). Therefore, these two PC axes accounted for major components of their niche dynamics in two dimensions. The AUC values for the 18 baseline ENMs, which were developed to project their potential ranges in native and introduced regions, fell within the range of 0.901 to 0.984 and 0.939 to 0.986, with mean values of 0.935 ± 0.024 and 0.967 ± 0.015, respectively (Table S8). Similarly, the TSS values in the models of the 18 ant species in native and introduced regions ranged between 0.841 and 0.976 and between 0.787 and 0.982, averaging 0.928 ± 0.037 and 0.899 ± 0.059, respectively (Table S8). These consistently high AUC and TSS values confirm the high reliability and accuracy of our ENMs.

### 3.2 Factors Influencing Niche and Range Shifts

**FIGURE 2:**
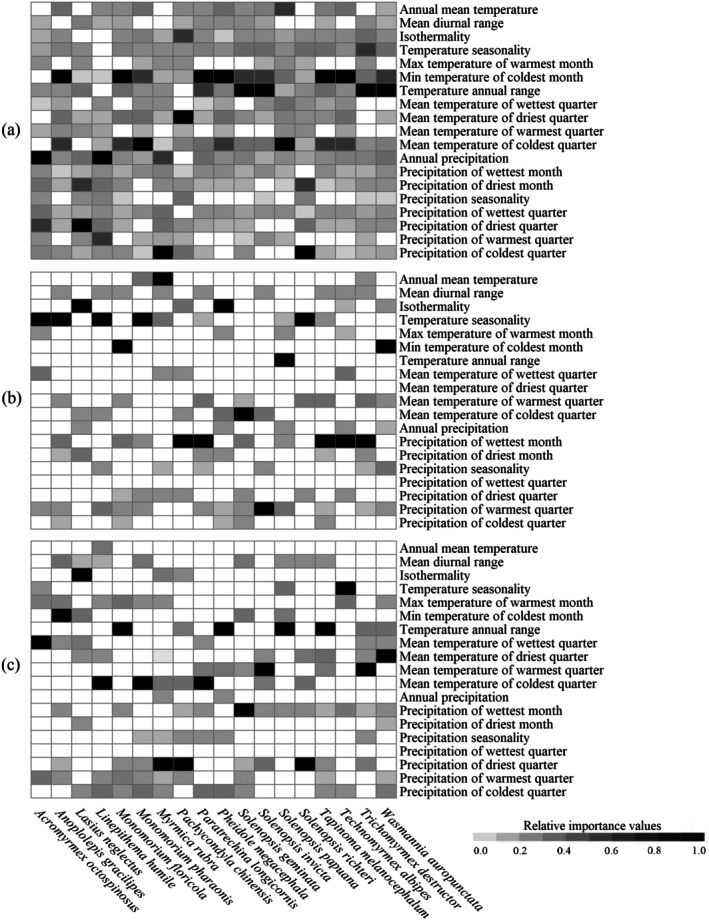
Variable importance in niche and range dynamic models. We standardized importance values through the maximum‐minimum method. In this figure, a–c indicate variable importance in the niche models and range dynamic models of native and introduced populations, respectively.

The predominant predictors of the niche dynamics varied among species (Figure 2a and Table S9). For example, annual precipitation was the predictor (0.896) with the highest eigenvalue for explaining variation in the niches between introduced and native populations of *Acromyrmex octospinosus* , and the most important predictor of niche shifts between introduced and native populations of *Paratrechina longicornis* was min temperature in the coldest month (0.897) (Table S9). Overall, min temperature in the coldest month was the most significant predictor explaining variation in the niche dynamics of six ant species, followed by temperature annual range for four species (Figure 2a). Additionally, temperature‐related predictors explained the most variation in the niche dynamics of the most significant invasive ant species (15/18) (Figure 2a).

The predominant predictors responsible for the potential ranges of the 18 major invasive ant species were species‐specific (Figure 2b,c and Table S9). For example, for the potential ranges of *Myrmica rubra* and *Pachycondyla chinensis* in native regions, the most important predictors were mean annual temperature and precipitation in the wettest month, respectively (Figure 2b). The main predictors for *Pheidole megacephala* and *Solenopsis invicta* in the introduced regions were annual temperature range and averaged temperature in the warmest season, respectively (Figure 2c). Additionally, the main predictors of the ranges also varied between the introduced and native counterparts for most invasive ant species. For example, averaged temperature in the coldest season was the predominant predictor of the native range of *Solenopsis geminata* , while that for the introduced range was precipitation in the wettest month. Overall, both temperature seasonality and precipitation in the wettest month were the major predictors of the native potential ranges of five species, and annual temperature range and precipitation in the driest season were the major predictors of the introduced ranges of four and three species, respectively. Additionally, temperature‐related predictors were the predominant predictors responsible for 66.7% and 77.7% of the ranges of the 18 species in native and introduced regions (Figure 2b,c).

### 3.3 Potential Ranges of the Worst Invasive Ants

The MSSs that were used to calibrate the potential ranges varied among species. For example, the MSSs for the native and introduced ranges of *Acromyrmex octospinosus* were 0.790 and 0.735, respectively, while those for *Tapinoma melanocephalum* were 0.800 and 0.720, respectively (Table S10). The MSSs for the native and introduced populations of all species varied from 0.740 to 0.880, with an average being 0.800 ± 0.039 and from 0.650 to 0.875, with an average being 0.745 ± 0.051, respectively (Table S10).

**FIGURE 3:**
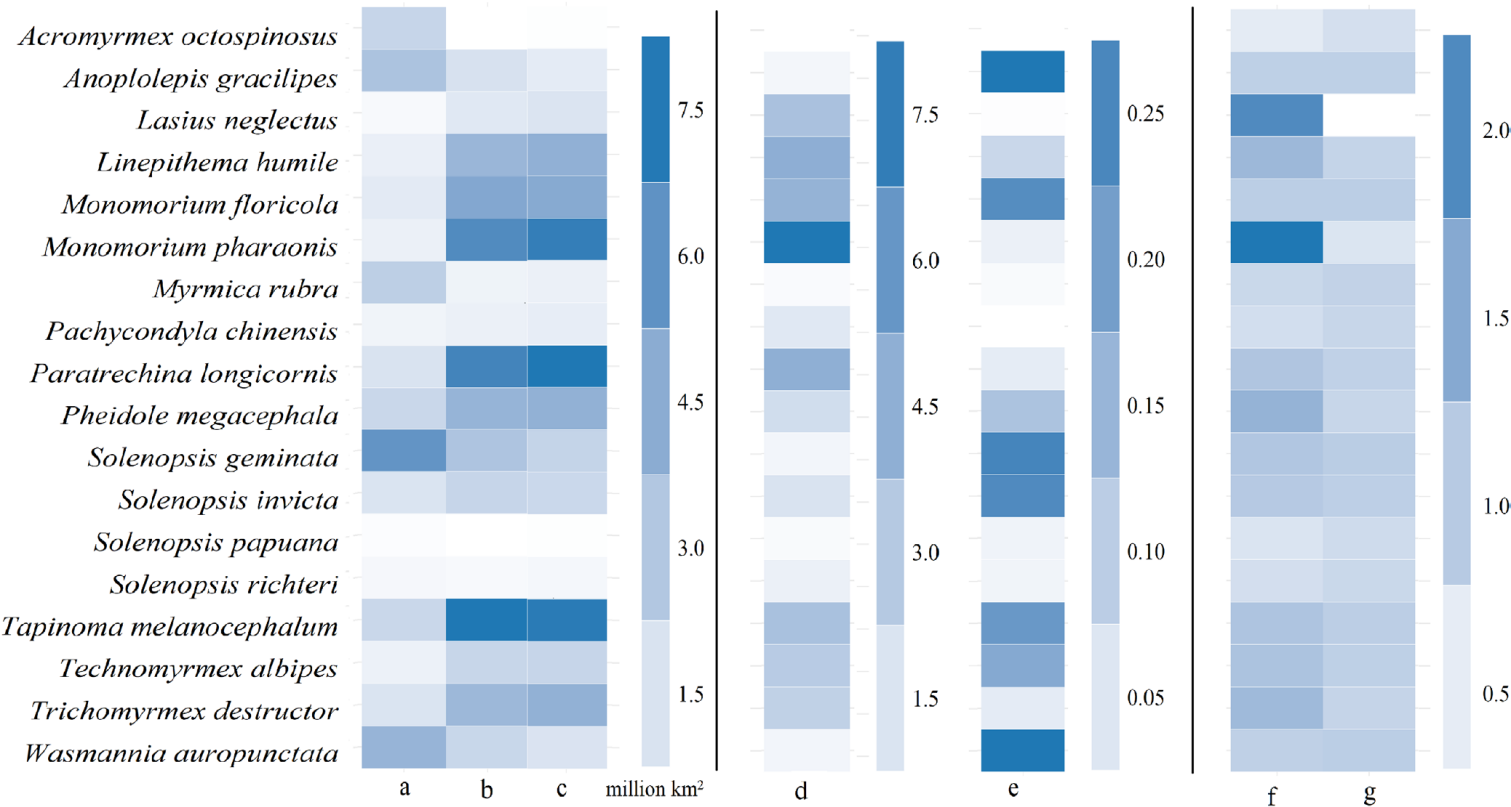
Major indices in range and niche dynamics. In this figure, a–c represented area of native, potential, and expanding ranges, respectively. We also used d–g to indicate range ratio index and range similarity index, niche breadth ratio and niche similarity index, respectively.

Although the ranges of most species were identified in tropical regions, spatial patterns of their ranges varied among species. For example, the potential ranges of *Trichomyrmex destructor* in native regions were primarily detected in India, while those of *Pachycondyla chinensis* were mainly projected in East China (Data S1). The range size also varied among species. For example, the ranges of all species in native ranges ranged from 0.23 million km^2^ ( *Solenopsis papuana* ) to 6.68 million km^2^ ( *Solenopsis geminata* ), and those in introduced regions ranged from 0.06 million km^2^ ( *Acromyrmex octospinosus* ) to 8.58 million km^2^ ( *Tapinoma melanocephalum* ) (Figure 3a,b). *Solenopsis papuana* in its introduced regions covered a potential range of 0.07 million km^2^, while that for *Lasius neglectus* covered 3.46 million km^2^ (Figure 3b). Additionally, the size and spatial patterns of their potential ranges showed substantial divergence between native populations and their introduced counterparts. For example, the potential range of *Myrmica rubra* in the native regions included Europe, covering 2.97 million km^2^, while that for its introduced counterparts included the eastern part of North America and covered 0.8 million km^2^ (Figure 3a,b). We also noted that 38.8% of our target species in their introduced regions were projected to have smaller potential ranges than those in native regions (Table 1).

**TABLE 1:** Testing niche and range dynamic conservatism.

Species	TPSR	BR	NSI	NC tests	RR	RSI	RC tests	BR < RR	NSI > RSI
*Acromyrmex octospinosus* Reich	0.063	0.522	0.686	Y	0.022	0.000	Y	N	Y
*Anoplolepis gracilipes* Smith	0.997	0.925	0.923	Y	0.495	0.285	Y	N	Y
*Lasius neglectus* Van Loon, Boomsma & Andrásfalvy	4.632	1.995	0.225	N	3.462	0.003	N	Y	Y
*Linepithema humile* Mayr	1.745	1.271	0.864	Y	4.543	0.080	N	Y	Y
*Monomorium floricola* Jerdon	2.406	0.955	0.952	Y	4.248	0.238	N	Y	Y
*Monomorium pharaonic* Linnaeus	3.531	2.290	0.608	Y	7.837	0.030	N	Y	Y
*Myrmica rubra* Linnaeus	0.756	0.798	0.876	Y	0.269	0.010	Y	N	Y
*Pachycondyla chinensis* Emery	1.171	0.715	0.831	Y	1.289	0.000	N	Y	Y
*Paratrechina longicornis* Latreille	1.579	1.068	0.896	Y	4.490	0.038	N	Y	Y
*Pheidole megacephala* Fabricus	1.604	1.355	0.834	Y	1.908	0.122	N	Y	Y
*Solenopsis geminata* Fabricus	0.956	1.060	0.956	Y	0.540	0.256	Y	N	Y
*Solenopsis invicta* Buren	1.663	1.013	0.932	Y	1.539	0.244	N	Y	Y
*Solenopsis papuana* Emery	0.738	0.619	0.752	Y	0.308	0.024	Y	N	Y
*Solenopsis richteri* Forel	0.635	0.705	0.802	Y	0.835	0.022	Y	Y	Y
*Tapinoma melanocephalum* Fabricius	2.169	1.088	0.938	Y	3.481	0.214	N	Y	Y
*Technomyrmex albipes* Smith	2.199	1.112	0.917	Y	2.879	0.176	N	Y	Y
*Trichomyrmex destructor* Jerdon	1.498	1.245	0.847	Y	2.582	0.039	N	Y	Y
*Wasmannia auropunctata* Roger	1.642	0.909	0.949	Y	0.537	0.285	Y	N	Y

Abbreviations: BR, niche breadth ratios; NC tests, niche conservatism tests; NSI, niche similarity indices; RC tests, range conservatism tests; RR, range ratios; RSI, range similarity indices; TPSR, top predictor span ratios.

**FIGURE 4:**
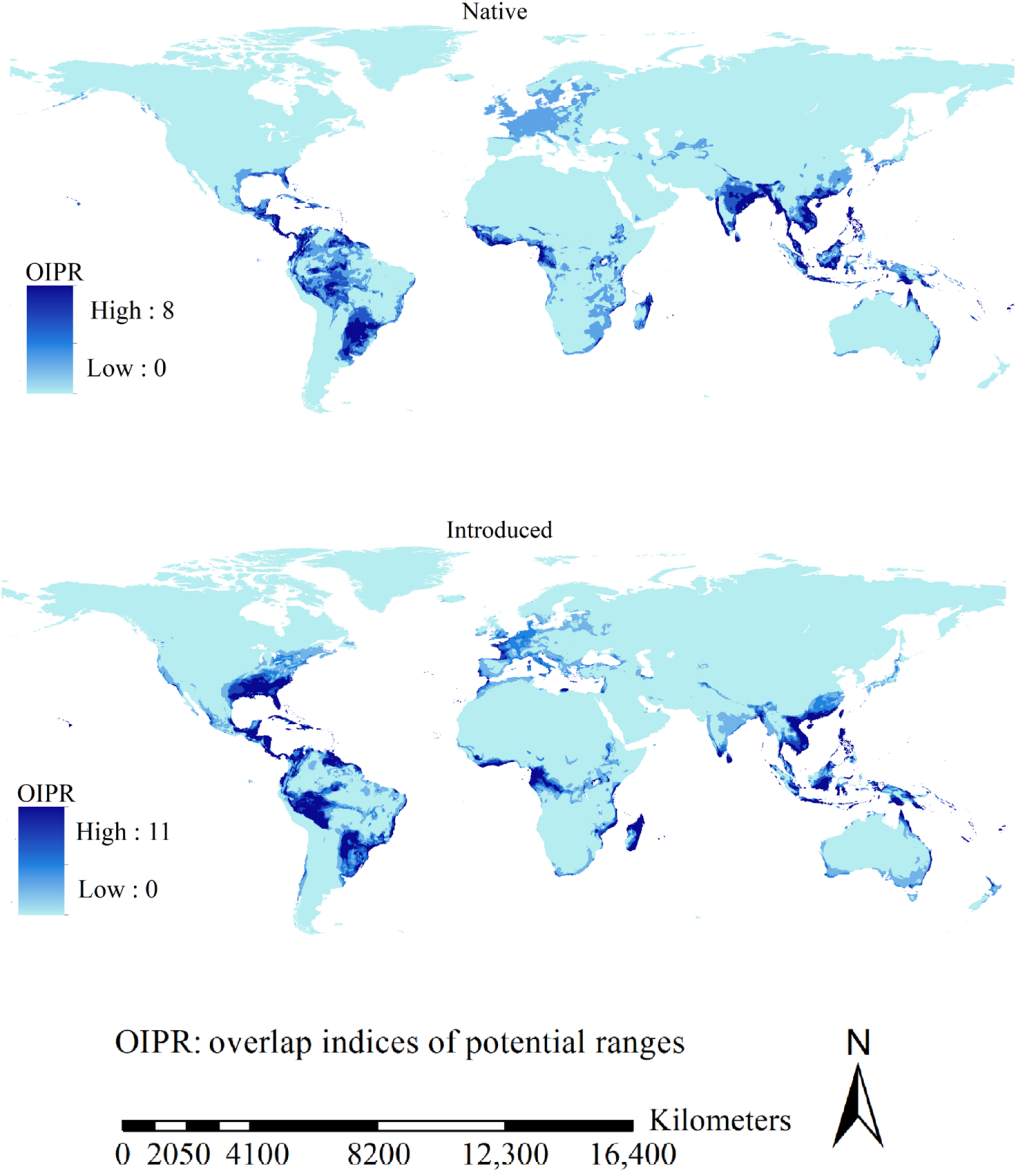
Range overlap of the invasive ants in native and introduced regions. Range overlap of the native populations was high in tropical regions, except for India; high range overlap of the introduced populations also included the southeastern USA and small regions in Europe.

High range overlap of the 18 native populations was primarily identified in tropical regions in Asia, Africa, and America, except for India (Figure 4a). High range overlap of the introduced populations included the southeastern USA and small regions in Europe (Figure 4b).

### 3.4 Niche Dynamics of Major Invasive Ants

**FIGURE 5:**
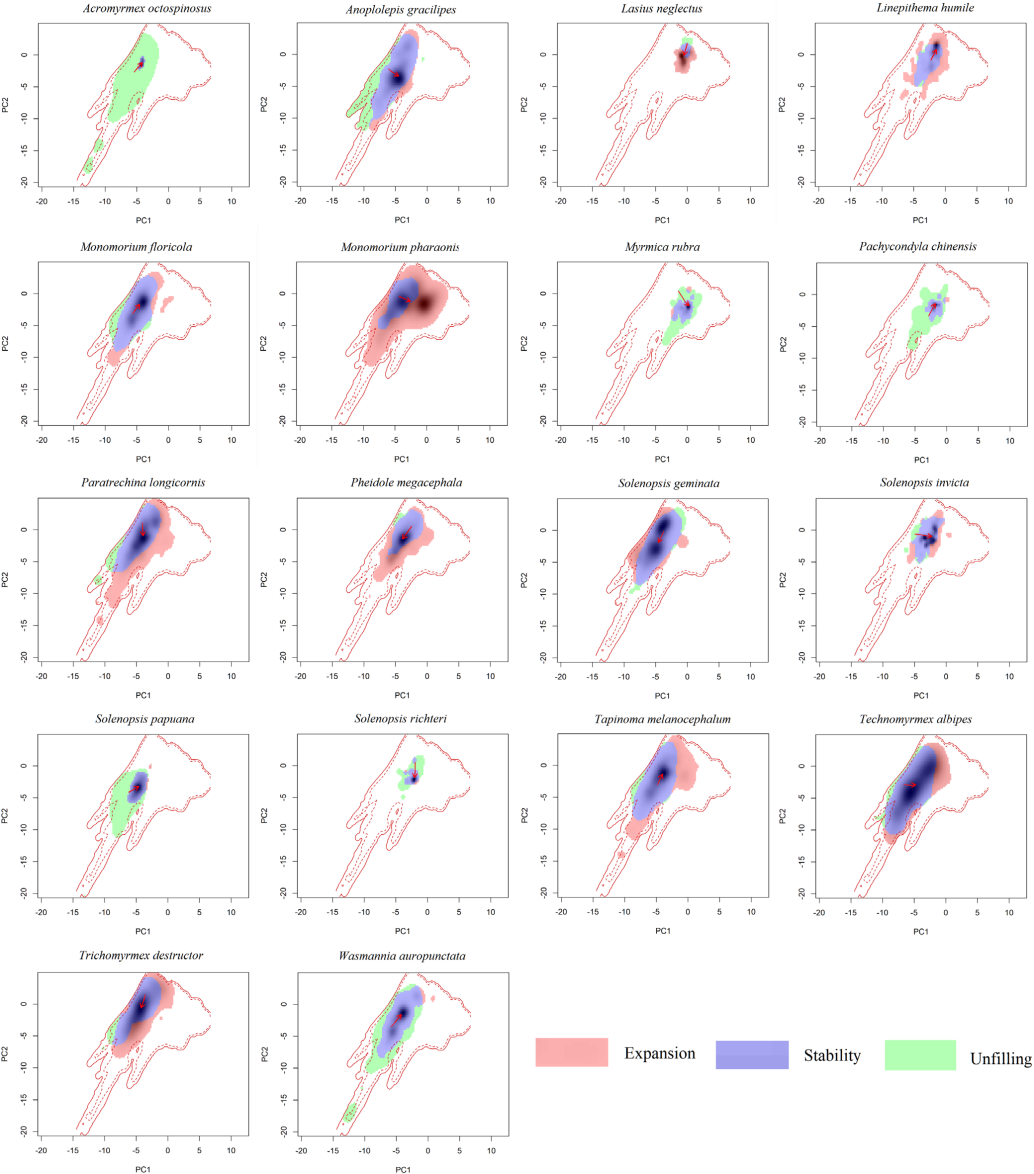
Niche dyamics of major invasive ant species. Ember, blue and green represented niche expansion, niche stability and niche unfilling, in this order.

The niche dynamics varied among our target species (Table 1 and Figure 5). For example, the niche breadth ratios ranged from 0.522 ( *Acromyrmex octospinosus* ) to 2.290 ( *Monomorium pharaonis* ) with 10 of the 18 ant species showing wider introduced niche breadths than native ones (Table 1 and Figures 3 and 5). The niche similarity indices varied from 0.225 (Lasius neglectus) to 0.956 ( *Solenopsis geminata* ) with only one of them being < 0.500 (*Lasius neglectus*). Only one species (*Lasius neglectus*) had niche breadth ratios > 1.000 and niche similarity indices smaller than 0.500; hence, only one species rejected the niche conservatism hypothesis (Table 1 and Figures 3 and 5).

### 3.5 Range Dynamics and Their Associations With Niche Dynamics

The range shifts depended on species. For example, range ratios ranged from 0.02 ( *Acromyrmex octospinosus* ) to 7.84 (*Monomorium pharaonic*), and range similarity indices ranged from 0.00016 ( *Acromyrmex octospinosus* ) to 0.285 ( *Anoplolepis gracilipes* ) (Figure 4). A total of 7 of the 18 target species in the introduced regions were projected to have smaller potential ranges than their native counterparts (Table 1 and Figure 3). All 18 range similarity indices were < 0.5 (Table 1 and Figure 3). Therefore, the range conservatism hypothesis did not apply to 11 of the 18 target species (Table 1).

**FIGURE 6:**
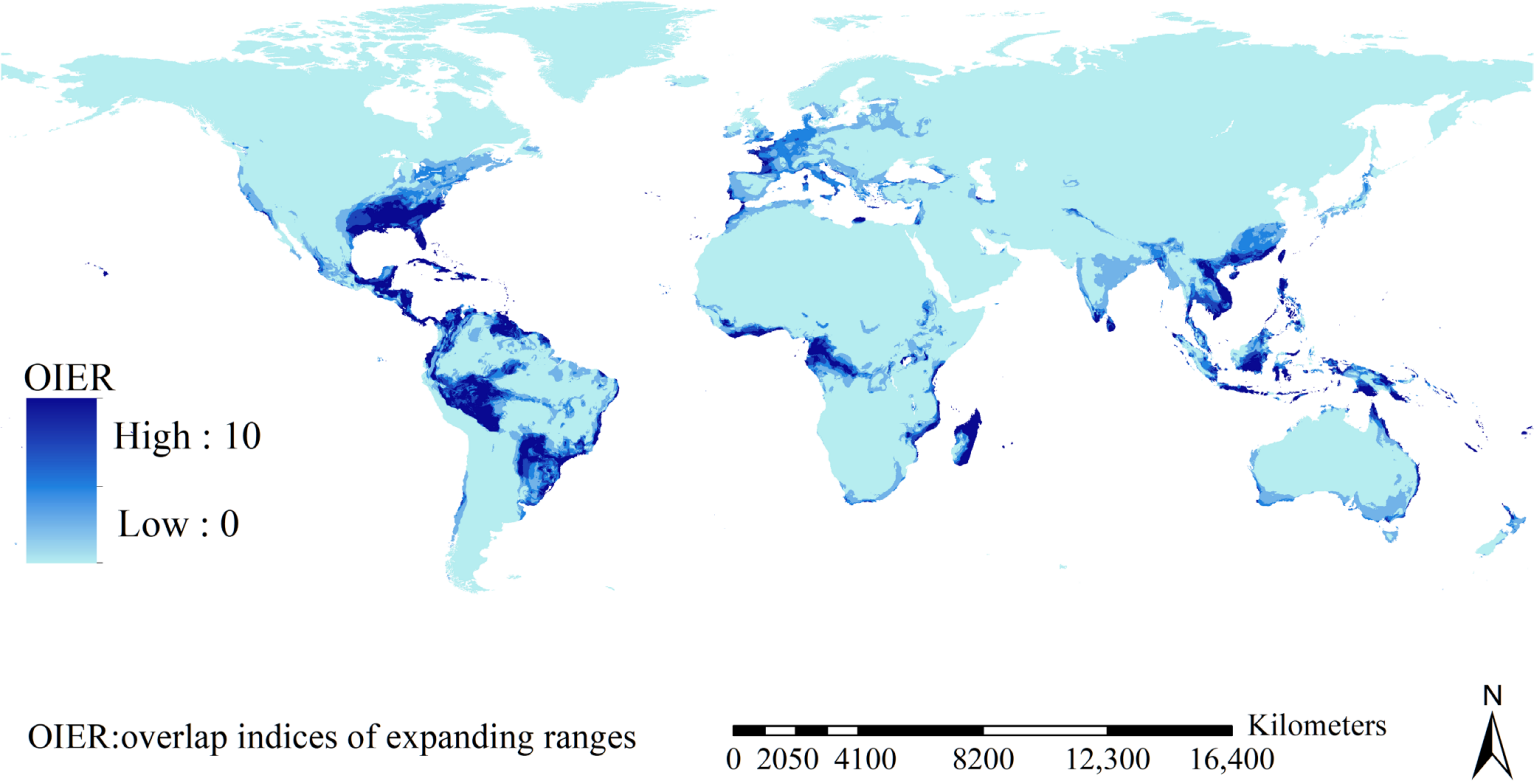
Expanding range overlap of major invasive ant species. Expanding ranges were mostly projected in the southeastern USA, Mexico, Brazil, Chile, Peru, Madagascar, Gabon, Republic of the Congo, tropical regions of Asia, and a small part of Europe.

The expanding ranges, which represented the ranges potentially occupied by the introduced populations but not by their native counterparts, were mostly projected in the southeastern USA, Mexico, Brazil, Chile, Peru, Madagascar, Gabon, Republic of the Congo, tropical regions of Asia, and a small part of Europe (Figure 6). Additionally, the sizes of the expanding ranges ranged from 0.06 million km^2^ ( *Acromyrmex octospinosus* ) to 7.57 km^2^ ( *Paratrechina longicornis* ) (Figure 3).

**FIGURE 7:**
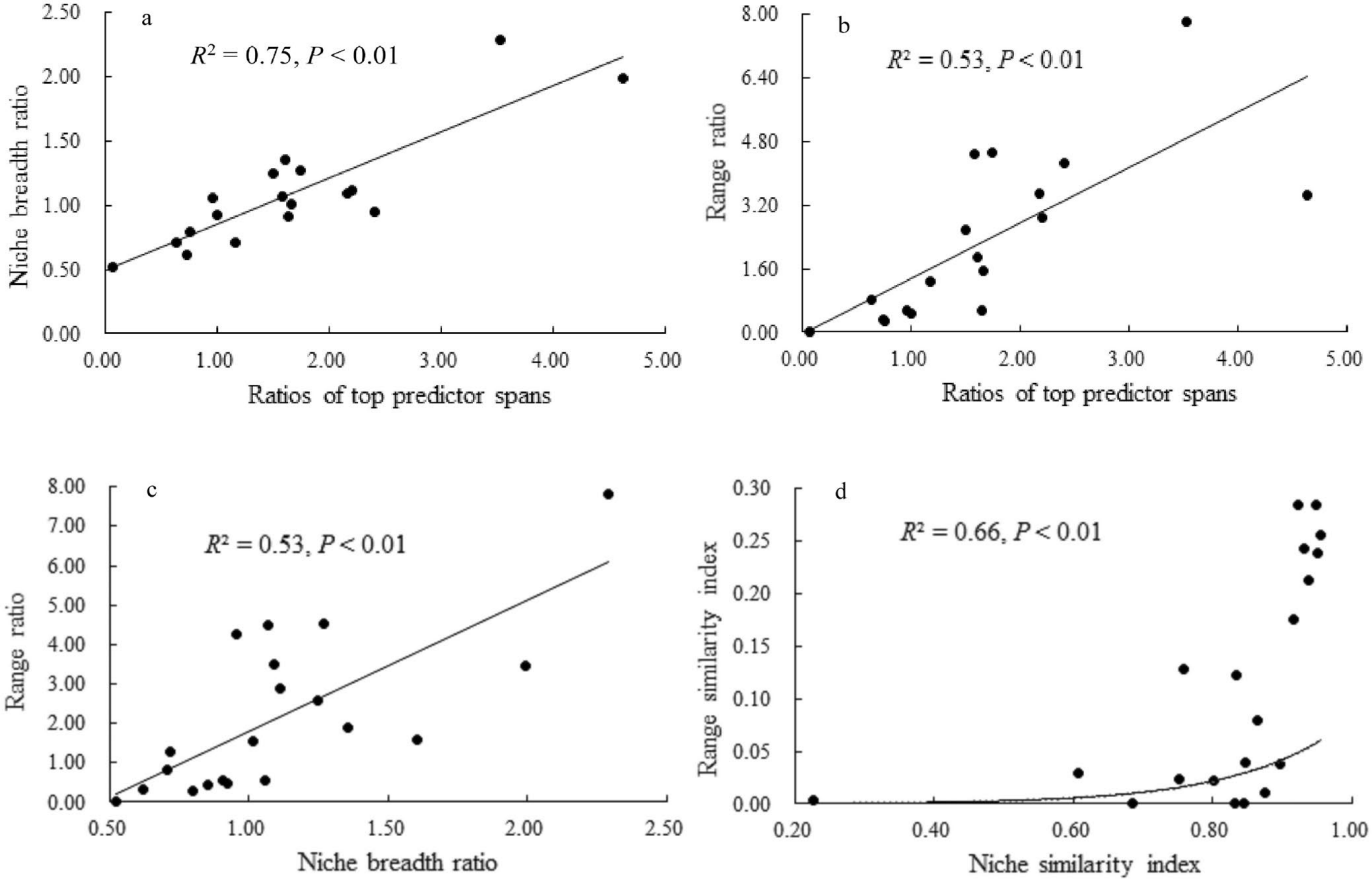
The relationship between niche and range dynamics, and their associations with predictor ranges. In this figure, a indicated the positive and significant associations between niche breadth ratios and ratios of top predictors ranges; b indicated the positive and significant associations between range ratios and ratios of top predictors ranges; c represented the positive and significant associations between niche breadth ratios and range ratios; d showed the positive and significant associations between niche similarity indices and range similarity indices.

Paired sample *t*‐tests showed that when niche breadth ratios were > 1.000, the range ratios were greater than the niche breadth ratios (3.326 vs. 1.350, *p* = 0.007), but this was not the case when niche breadth ratios were < 1.000 (0.937 vs. 0.768, *p* = 0.708) (Table 1 and Figure 3). Paired sample *t*‐tests indicated that range similarity indices were significantly smaller than niche similarity indices (0.115 vs. 0.822, *p* = 0.001). Additionally, our study showed that 90% and 100% of species showing niche and range expansions could survive in climatic conditions with larger ranges of the main predictors, respectively (Table 1). Our findings also indicated that there were positive and significant associations between niche breadth ratios and range ratios, between niche similarity indices and range similarity indices, as well as between niche breadth ratios, range ratios, and the ratios of the predictor ranges (Figure 7).

## 4 Discussion

Invasive ants have major effects on global ecosystems and the economy. Using 133,786 global occurrence records, we developed niche and range dynamic models for each of 18 IUCN‐recognized major invasive ant species and then examined their climatic niches and range shifts between their introduced and native populations. The findings indicated that most of our introduced species populations showed substantial niche and range expansions relative to their native populations. In other words, we assumed that most of the target species in introduced/invaded regions are highly adapted to novel climatic conditions and could survive under more diverse climatic conditions than species in native regions. However, we were not sure whether their adaptation had taken place or not because only the shifts of realized niche were examined in our study. Additionally, our study is the first to comprehensively examine the niche and range dynamics of the native and introduced populations of all 18 IUCN‐recognized major invasive ant species within a single framework, which enables direct comparisons of the range and niche shifts among species. Moreover, we assessed the overlap of their expanding ranges and ranges to identify priority regions of their invasions. Overall, our research provides valuable insights that enhance our understanding of the range and niche dynamics of the worst invasive ant species and has implications for mitigating their impacts on the global economy and the ecosystems.

Several studies have probed their range shifts under future climate change scenarios (e.g., Chen 2008; Chifflet et al. 2018; Li et al. 2023, 2024). We noted that most of them focused on the changes in the range sizes of several invasive ant species and the regions where their ranges potentially occur under current–future scenarios. However, in our study we created two quantitative indicators, that is, range ratio index and range similarity index, to quantitatively calibrate their range shifts. Specifically, we used range ratio index to quantitatively measure the range expansions of all 18 IUCN‐recognized invasive ant species that could be adopted to assess their invasion risk relative to each other. Additionally, we used range similarity index to calibrate the range position shifts in all 18 IUCN‐recognized invasive ant species that were used to quantitatively assess the shifts in the priority regions where the invasive ant species could invade. Therefore, compared with those previous studies, our study provided a more comprehensive and quantitative characterization of the range shifts in major invasive ant species.

Our study revealed that introduced *Tapinoma melanocephalum* and *Paratrechina longicornis* had the largest potential ranges and expanding ranges, respectively, suggesting that they had higher invasion risks compared with other species based on their range sizes. Additionally, *Monomorium pharaonic* had the largest range ratios and niche breadth ratios among the 18 major invasive ant species. Therefore, *Tapinoma melanocephalum* , *Paratrechina longicornis* , and *Monomorium pharaonic* had the largest introduced range and niche expansion ratios relative to their native counterparts. The lowest range similarity indices and niche similarity indices were observed for *Acromyrmex octospinosus* , which indicated that it showed larger range position shifts in its ranges and niches relative to its native counterparts. We also noted that *Monomorium pharaonic* and *Acromyrmex octospinosus* , who showed substantial niche and range shifts, had smaller native ranges relative to others (Figure 3a), which, to a certain extent, was echoed by the findings of Bates et al. (2020) that the species with smaller native niches and ranges could experience larger niche shifts.

Five of our 18 invasive ant species were included in the latest list of the 100 World's Worst Invasive Alien Species, including *Linepithema humile* , *Anoplolepis gracilipes* , *Solenopsis invicta* , *Pheidole megacephala* , and *Wasmannia auropunctata* (Invasive Species Specialist Group 2025). In other words, according to the criteria of the Invasive Species Specialist Group (ISSG) of the IUCN, these are the world's most deleterious invasive species. However, in our study none of them were the species with the largest ranges, expanding ranges, niche and range expansion ratios, and the shifts in niche and range positions (Table 1 and Figure 3). Although they did not show more substantial shifts in niches and ranges than other species, this does not imply that they could not have larger effects on the global ecosystem and economy. Therefore, assessing the effects or invasiveness of the major invasive ant species requires extensive studies, and studies of niche and range shifts will be critical for assessing their invasion risk.

Invasive species often exhibit broader climatic niches and potential ranges when they are introduced to new environments, suggesting that they can adapt to conditions that differ from those in their native habitats (Cao et al. 2022; Wu et al. 2023). Our research detected substantial niche and range expansions in most of the 18 major invasive ant species in introduced regions. This demonstrates that most of our target species had a strong capacity to adjust to novel environmental conditions and highlights their high potential for invasion into regions in which climatic conditions differ from those of their original ranges. These niche and range expansions might not only be closely associated with their strong adaptability to novel climatic conditions (Lach 2021; Parr and Bishop 2022) but also with intentional or unintentional human‐mediated introductions, such as invasive ants in the United States (Lofgren 2019), where the environmental conditions differ considerably from those of their native areas. These introductions likely helped most of the introduced populations overcome natural dispersal barriers and provided more opportunities for the species to thrive in novel climate conditions compared with most of the native populations (Lofgren 2019; Li et al. 2023), which were echoed by the findings of Cao et al. (2022) and Wu et al. (2023). For most of the introduced populations, this process probably could enhance their exploitation of intrinsic adaptability to diverse climate conditions. This argument is supported by our observations that most of the target species showing wider niche breadths and larger ranges in the introduced regions than in native regions could withstand larger climate variability represented by the top predictor ranges than those in native regions; this is also consistent with the positive and significant associations between niche breadth ratios, range ratios, and the ratios of the top predictor ranges. However, our study demonstrated that 8 and 7 of the 18 target species' introduced populations showed niche and range contractions (unfilling) relative to their native populations, respectively. Therefore, these niche and range expansions/contractions might be species‐specific, which probably depends on their capacity to survive in novel environmental conditions and the strength of human‐mediated introductions; additional studies are needed to clarify these possibilities.

Invasive ant species have major effects on global ecosystems and economy (Gruber et al. 2021, 2022; Siddiqui et al. 2021). Regions with higher range overlap among invasive ant species might experience stronger and more complicated impacts compared with other regions. Our findings revealed that substantial introduced range overlap was mainly identified in the southeastern USA and small regions in Europe. The expanding ranges, which represented the ranges solely occupied by the introduced populations, were mostly projected in the southeastern USA, Mexico, Brazil, Chile, Peru, Madagascar, Gabon, Republic of the Congo, tropical regions of Asia, and a small part of Europe. This implies that these regions merit increased attention for mitigating the impacts of the invasions of these major invasive ants.

We showed that only one of the 18 species rejected the niche conservatism hypothesis; this observation has been supported for most invasive species as evidenced by Liu et al. (2020). However, our findings indicated that the range conservatism hypothesis did not apply to most of the major invasive ant species, given that most of them have undergone substantial range shifts. Therefore, niche and range dynamics might be controlled by different underlying factors, albeit additional investigation is needed to clarify this possibility. Additionally, their small niche shifts did not necessarily suggest that they did not have high invasion risks because we detected substantial range shifts between the introduced and native populations. The distribution of species across geographic regions is frequently tied to their niche or their ability to utilize available resources. Although numerous studies have identified positive relationships between a species' niche breadth and the size of its range, this pattern is not universally consistent, as indicated by various studies (Slatyer et al. 2013; Moore et al. 2018; Bernard et al. 2021; Kafaei et al. 2021). This inconsistency underscores the need for more studies that examine the interplay between niche dynamics and range changes, especially in the context of biological invasions. Our research demonstrated that there were significant and positive associations between introduced and native niches and ranges, including between niche and range ratio indices and niche and range similarity indices. Of note, the close and positive relationship between niche and range similarity indices implied that large niche position shifts generally resulted in huge range position shifts, and vice versa, which, to a certain extent, was supported by the findings of Wu et al. (2023). Additionally, most species have undergone range expansions, and most of their range ratios were higher than their niche breadth ratios (Table 1), implying that small niche expansions could bring about substantial range expansions. Our findings also showed that niche similarity indices were significantly smaller than range similarity indices, suggesting that small shifts in their niche positions could induce large range position shifts. Additionally, we found that the hypotheses of niche and range conservatism were rejected by one and 11 of the 18 target species, respectively. These findings implied that even small‐scale niche shifts can induce substantial shifts in a species' range, which was supported by the findings of Cao et al. (2022) and Wu et al. (2023). As a result, shifts in niche characteristics may act as highly sensitive predictors of potential range changes, highlighting their importance in combating invasive species. Although range shifts have often been adopted to evaluate invasive species invasions (Moore et al. 2018; Gong et al. 2020; Jung et al. 2022), our findings imply that niche shifts could offer more sensitive metrics for assessing the risks associated with biological invasions. For example, Wu et al. (2023) argued that small shifts in niche similarity indices might induce large centroid shifts in the Giant African Snail (*Lissachatina fulica*), suggesting substantial shifts in the priority regions against its invasions.

Of note, we took global terrestrial as our calibration area to explore the niche and range shifts of 18 invasive ant species, and did not consider the regions that have been accessible to our target species via dispersal over relevant periods of time. It, to a certain extent, resulted in biased pseudo‐absence generation and inflated model performance, and therefore undermined the model's ecological validity and reliability (Barve et al. 2011). We have to acknowledge that the spreading of invasive ants could also be strongly influenced by factors other than climatic ones, such as human activities (Bertelsmeier et al. 2017; Chifflet et al. 2018; Wong et al. 2023). However, our study focused solely on the niche and range dynamics of invasive ants driven by climate change. Therefore, caution is required when interpreting our observations.

## 5 Conclusions

Here, we designed the first unified scheme for examining global range and niche shifts between the introduced and native regions of 18 IUCN‐recognized major invasive ant species. We identified range and niche expansions in most of these species. Additionally, the niche conservatism hypothesis was rejected by a small part of the 18 target species, while the hypothesis of range conservatism was rejected by most of them. We also observed positive and significant associations between range and niche dynamics, and small niche shifts could induce large range shifts. Therefore, niche shifts provide a more sensitive indicator for evaluating the invasion risks of species compared with range shifts. We also examined spatial patterns of overlap of their ranges and expanding ranges, which revealed priority regions for mitigating their impacts. We compared their invasion risks based on their niche and range dynamics. Therefore, our findings provide novel and valuable insights for mitigating their impacts on global ecosystems and economic systems.

## Author Contributions

**Qiance Wei:** conceptualization (equal), formal analysis (lead), investigation (lead), methodology (equal), software (lead), validation (lead), visualization (lead), writing – original draft (equal), writing – review and editing (equal). **Xueyou Zhang:** formal analysis (supporting), software (supporting), writing – original draft (supporting), writing – review and editing (supporting). **Xiaokang Hu:** conceptualization (equal), funding acquisition (lead), methodology (equal), project administration (equal), supervision (equal), writing – original draft (equal), writing – review and editing (equal). **Jianmeng Feng:** conceptualization (equal), data curation (lead), methodology (equal), project administration (equal), resources (lead), supervision (equal), writing – original draft (equal), writing – review and editing (equal).

## Conflicts of Interest

The authors declare no conflicts of interest.

## Supporting information


**Table S1.** Invasive ant species in IUCN.

**Table S2.** List of literature and online datasets for retrieving occurrences.

**Table S3.** Importance values of the predictors in preliminary ENMs.

**Table S4.** Multi‐collinearity among predictors.

**Table S5.** Ten algorithms for building preliminary ENMs.

**Table S6.** Retained algorithms in baseline ENMs.

**Table S7.** PCA for niche dynamics.

**Table S8.** ENMs performance.

**Table S9.** Variable importance in niche and range dynamic models.

**Table S10.** MSS thresholds for determining ranges.

**Data S1.** Potential ranges and expanding ranges.

## Data Availability

The materials and datasets underlying this article could be retrieved in the Supporting Information.
