# Supplementary material for: Niche Shifts Induce Major Changes in the Ranges of the World's Worst Invasive Ant Species
Source: Ecol Evol. 2025 Jul 8;15(7):e71754. doi: 10.1002/ece3.71754 (PMC12237827; doi:10.1002/ece3.71754)
Supplement: Supplementary file 1 — Table S1. Invasive ant species in IUCN. [file ECE3-15-e71754-s011.docx]

**Table S1 List of major invasive ant species from IUCN**

| Common names | Scientific names | Families | Orders |
| --- | --- | --- | --- |
| Leaf-cutting ant | *Acromyrmex octospinosus* | Formicidae | Hymenoptera |
| Yellow crazy ant | *Anoplolepis gracilipes* | Formicidae | Hymenoptera |
| Invasive garden ant | *Lasius neglectus* | Formicidae | Hymenoptera |
| Argentine ant | *Linepithema humile* | Formicidae | Hymenoptera |
| Singapore ant | *Monomorium destructor* | Formicidae | Hymenoptera |
| Flower ant | *Monomorium floricola* | Formicidae | Hymenoptera |
| Pharaoh ant | *Monomorium pharaonis* | Formicidae | Hymenoptera |
| Red ant | *Myrmica rubra* | Formicidae | Hymenoptera |
| Asian needle ant | *Pachycondyla chinensis* | Formicidae | Hymenoptera |
| Long-legged ant | *Paratrechina longicornis* | Formicidae | Hymenoptera |
| Big-headed ant | *Pheidole megacephala* | Formicidae | Hymenoptera |
| Tropical fire ant | *Solenopsis geminata* | Formicidae | Hymenoptera |
| Red imported fire ant | *Solenopsis invicta* | Formicidae | Hymenoptera |
| Papuan thief ant | *Solenopsis papuana* | Formicidae | Hymenoptera |
| Black imported fire ant | *Solenopsis richteri* | Formicidae | Hymenoptera |
| Ghost ant | *Tapinoma melanocephalum* | Formicidae | Hymenoptera |
| White-footed ant | *Technomyrmex albipes* | Formicidae | Hymenoptera |
| Little fire ant | *Wasmannia auropunctata* | Formicidae | Hymenoptera |
| Caribbean crazy ant | *Nylanderia pubens* | Formicidae | Hymenoptera |
