# Supplementary material for: Niche Shifts Induce Major Changes in the Ranges of the World's Worst Invasive Ant Species
Source: Ecol Evol. 2025 Jul 8;15(7):e71754. doi: 10.1002/ece3.71754 (PMC12237827; doi:10.1002/ece3.71754)
Supplement: Supplementary file 2 — Table S2. List of literature and online datasets for retrieving occurrences. [file ECE3-15-e71754-s007.docx]

Table S2 List of major literature and online datasets for retrieving occurrence records of invasive ant species

**Major Literature**

| Booher DB, Gotelli NJ, Nelsen MP, Ohyama L, Deyrup M, Moreau CS, Suarez AV. Six decades of museum collections reveal disruption of native ant assemblages by introduced species. Current Biology. 2023 May 22;33(10):2088-94. |
| --- |
| Wong MK, Economo EP, Guénard B. The global spread and invasion capacities of alien ants. Current Biology. 2023 Feb 6;33(3):566-71. |
| Foucaud J, Orivel J, Loiseau A, Delabie JH, Jourdan H, Konghouleux D, Vonshak M, Tindo M, Mercier JL, Fresneau D, Mikissa JB. Worldwide invasion by the little fire ant: routes of introduction and eco‐evolutionary pathways. Evolutionary Applications. 2010 Jul;3(4):363-74. |
| Bertelsmeier C, Ollier S, Liebhold A, Keller L. Recent human history governs global ant invasion dynamics. Nature ecology & evolution. 2017 Jun 22;1(7):0184. |
| Bates OK, Ollier S, Bertelsmeier C. Smaller climatic niche shifts in invasive than non-invasive alien ant species. Nature Communications. 2020 Oct 15;11(1):5213. |
| Miravete V, Roura-Pascual N, Dunn RR, Gómez C. How many and which ant species are being accidentally moved around the world?. Biology letters. 2014 Aug 31;10(8):20140518. |
| Kass JM, Guénard B, Dudley KL, Jenkins CN, Azuma F, Fisher BL, Parr CL, Gibb H, Longino JT, Ward PS, Chao A. The global distribution of known and undiscovered ant biodiversity. Science advances. 2022 Aug 3;8(31):eabp9908. |
| Lee WH, Song JW, Yoon SH, Jung JM. Spatial evaluation of machine learning-based species distribution models for prediction of invasive ant species distribution. Applied Sciences. 2022 Oct 12;12(20):10260. |

**Major online datasets**

| Online datasets | Date of access |
| --- | --- |
| Global Biodiversity Information Facility | August 1^st^, 2024 |
| AntMaps | August 1^st^, 2024 |
| Global Ants Database | August 2^nd^, 2024 |
| AntWeb | August 1^st^, 2024 |
| Global Ant Biodiversity Informatics | August 2^nd^, 2024 |
| Antbase | August 3^rd^, 2024 |
