# Supplementary figures and images for: Niche Shifts Induce Major Changes in the Ranges of the World's Worst Invasive Ant Species
Source: Ecol Evol. 2025 Jul 8;15(7):e71754. doi: 10.1002/ece3.71754 (PMC12237827; doi:10.1002/ece3.71754)

S11Potential Ranges and expanding ranges


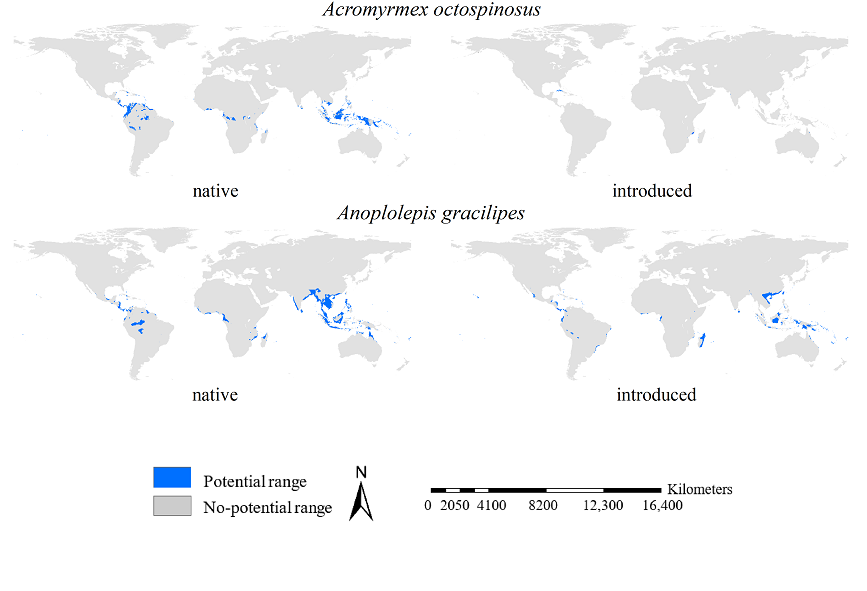


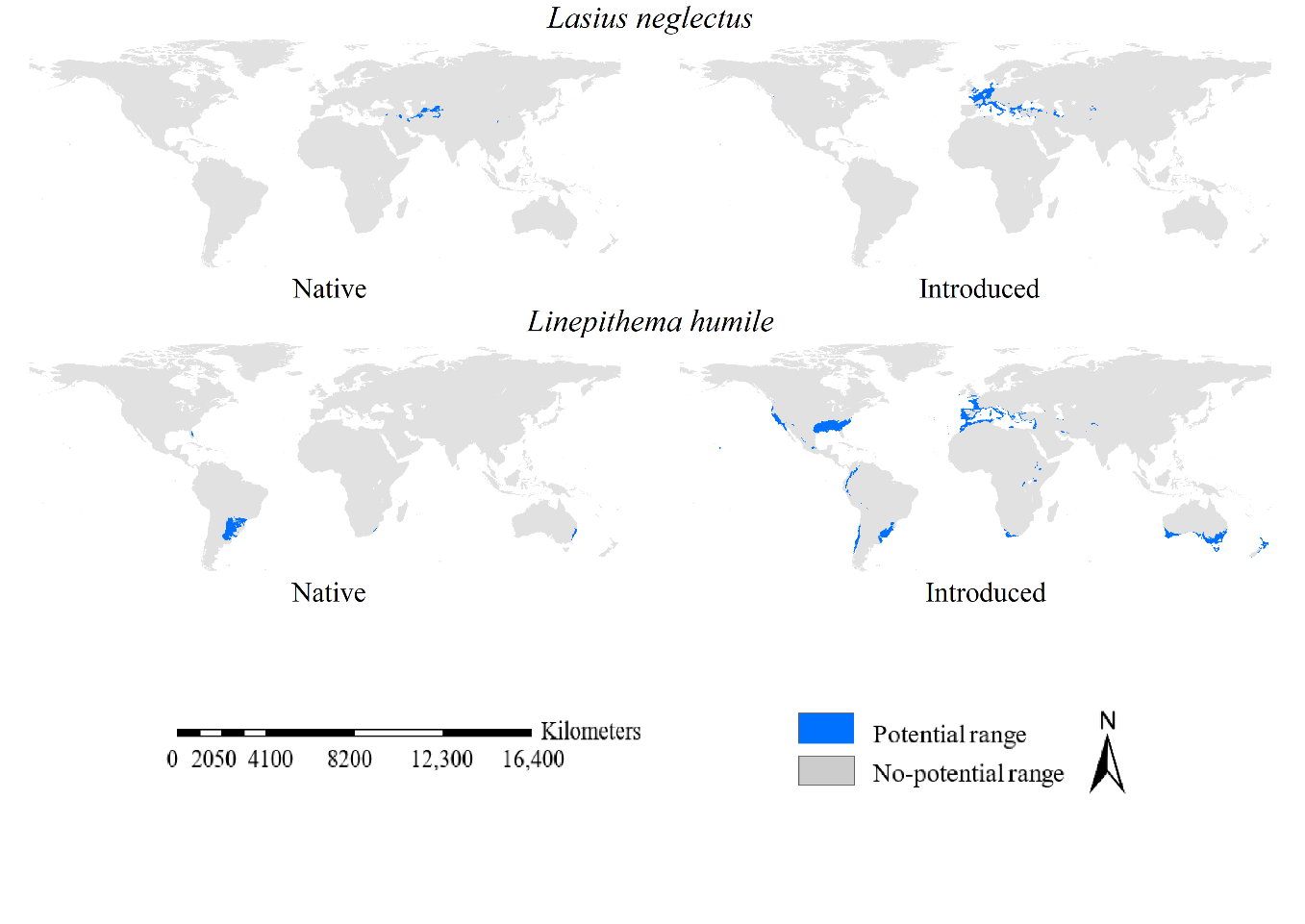


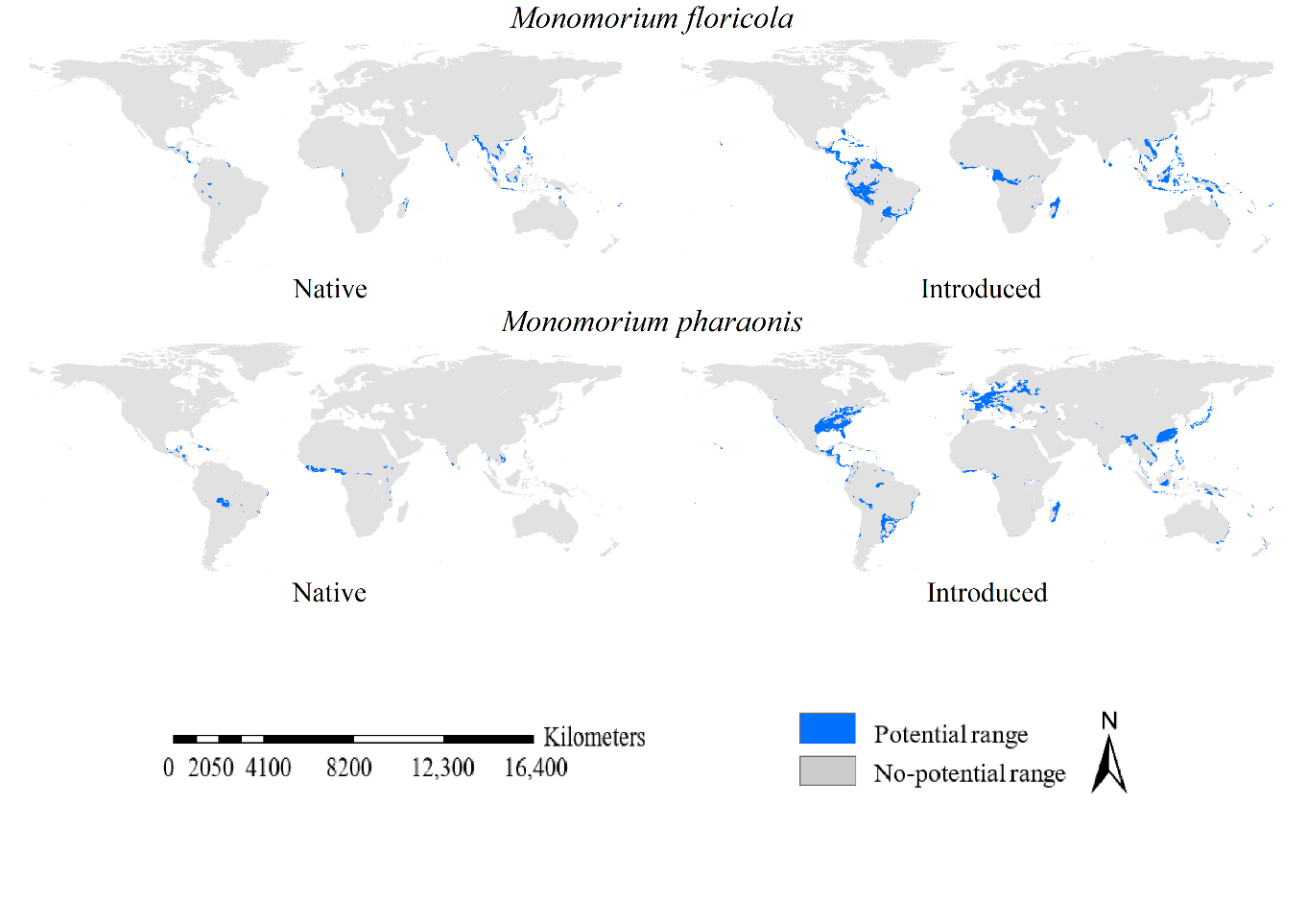


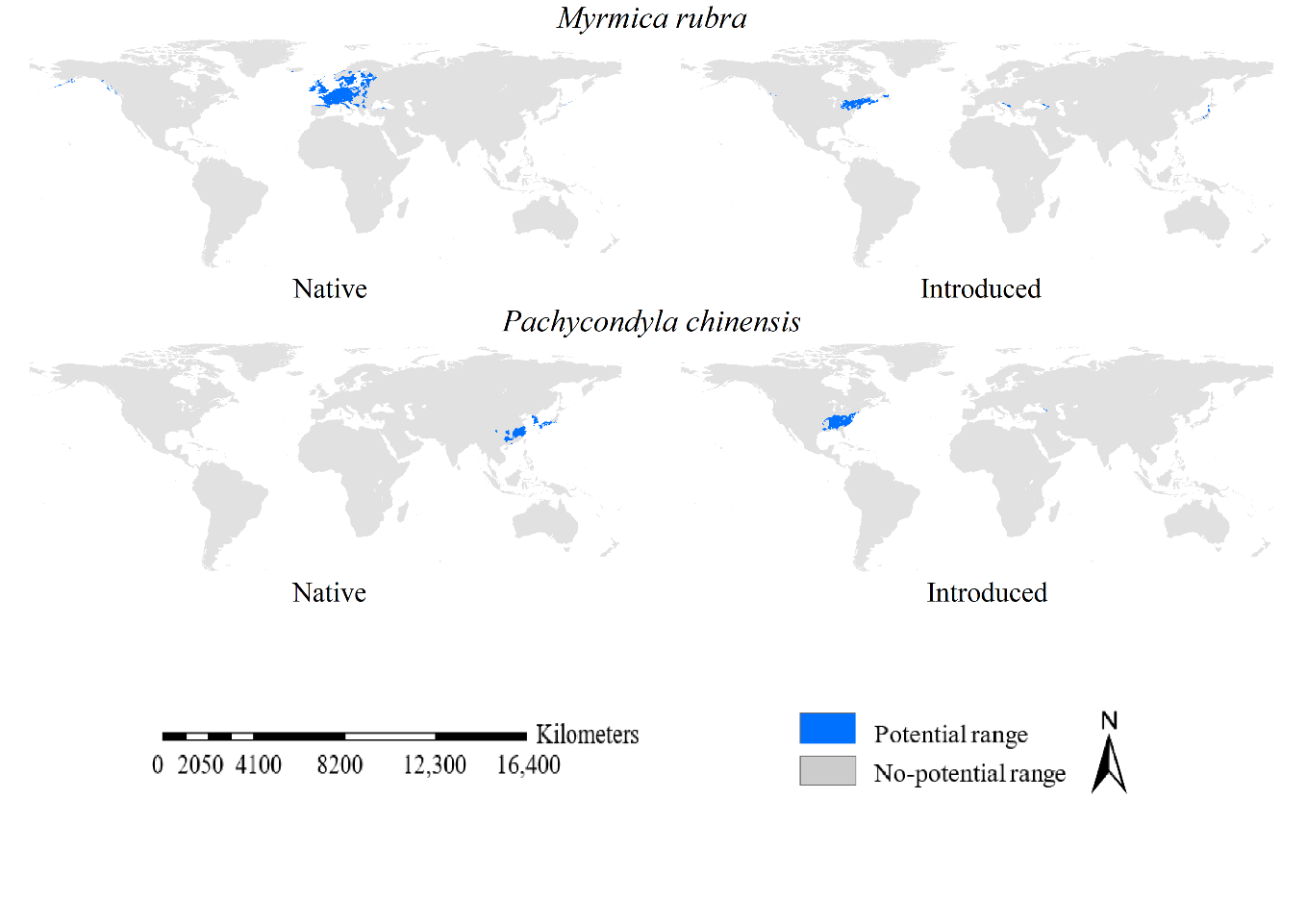


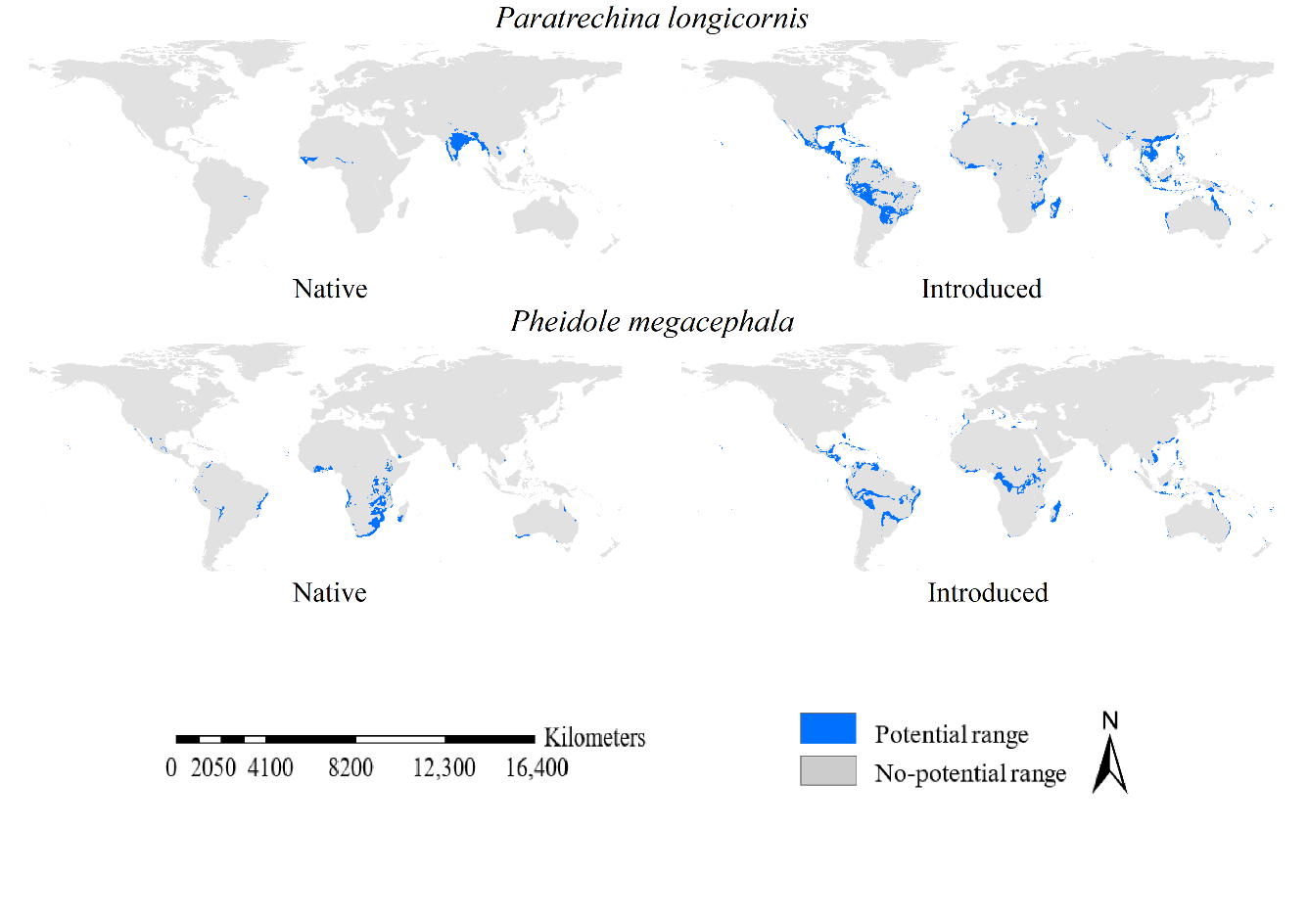


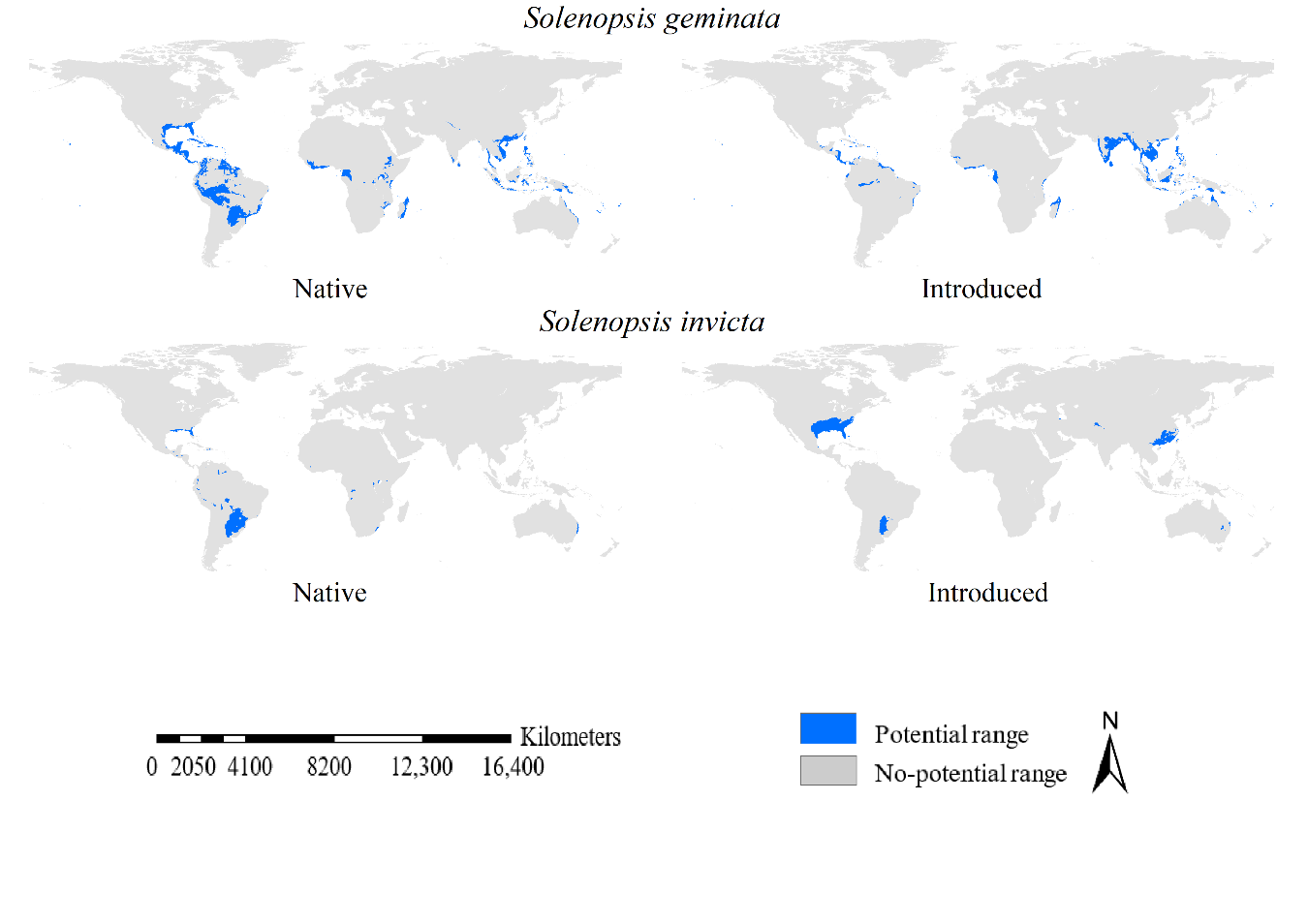


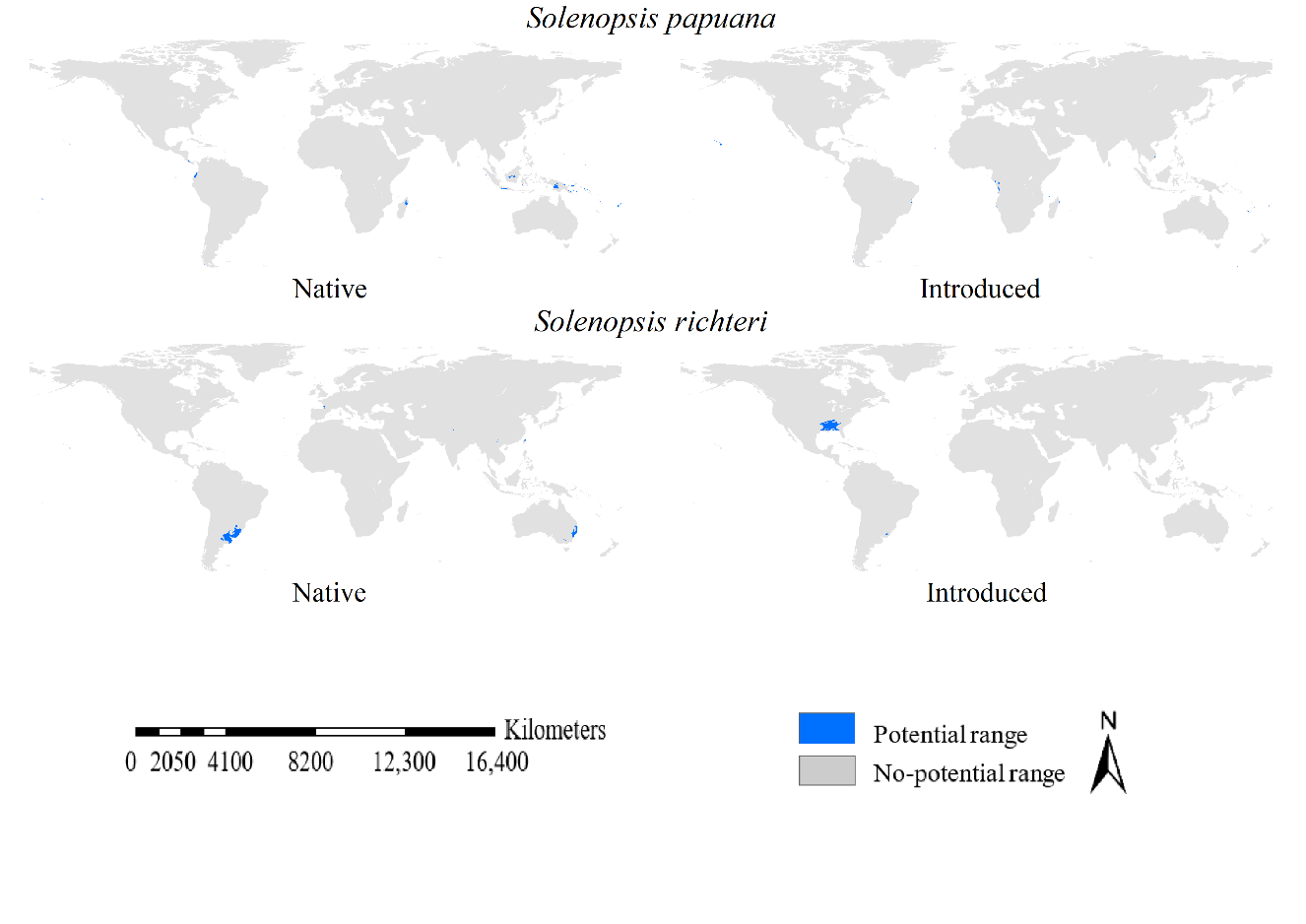


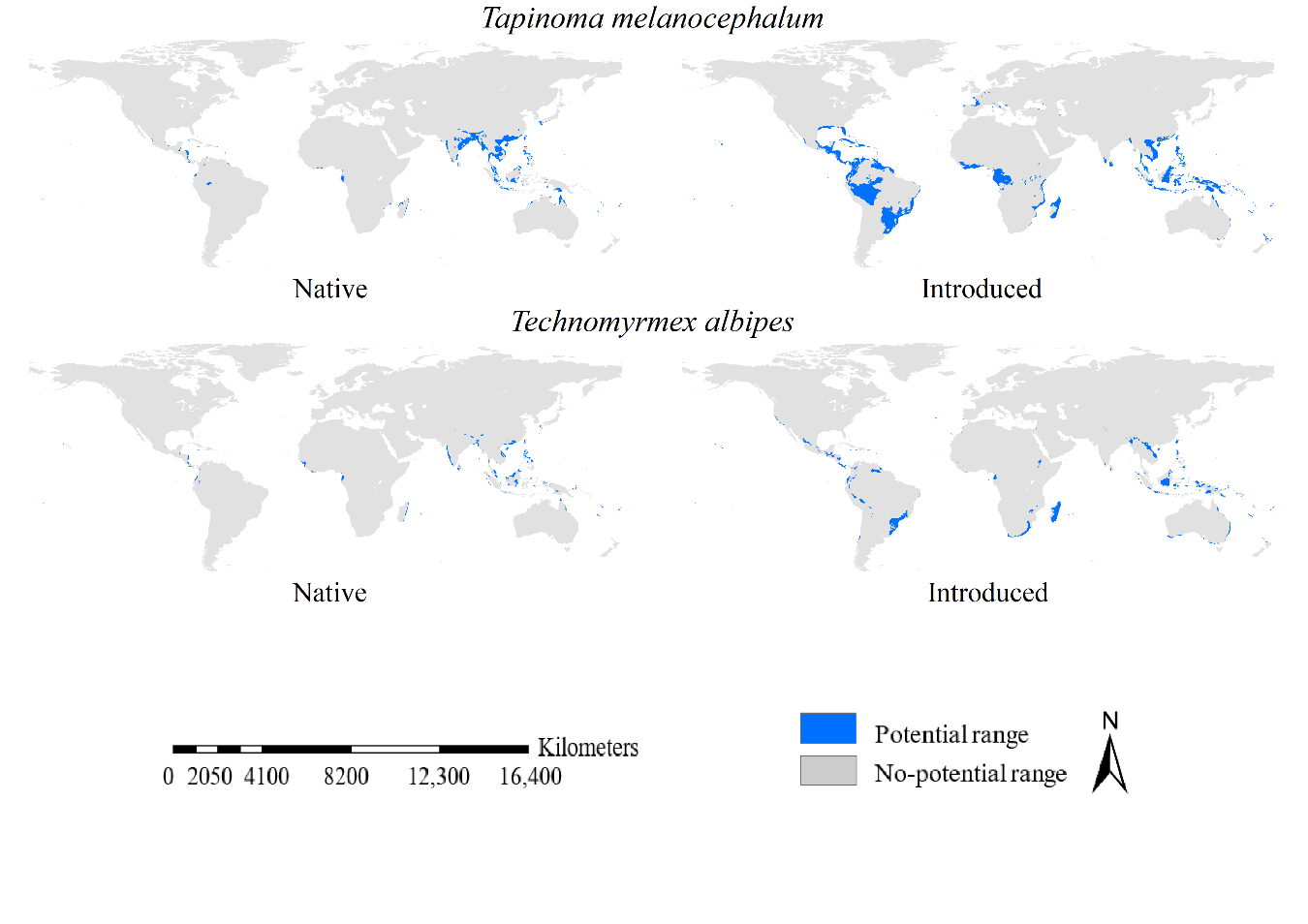


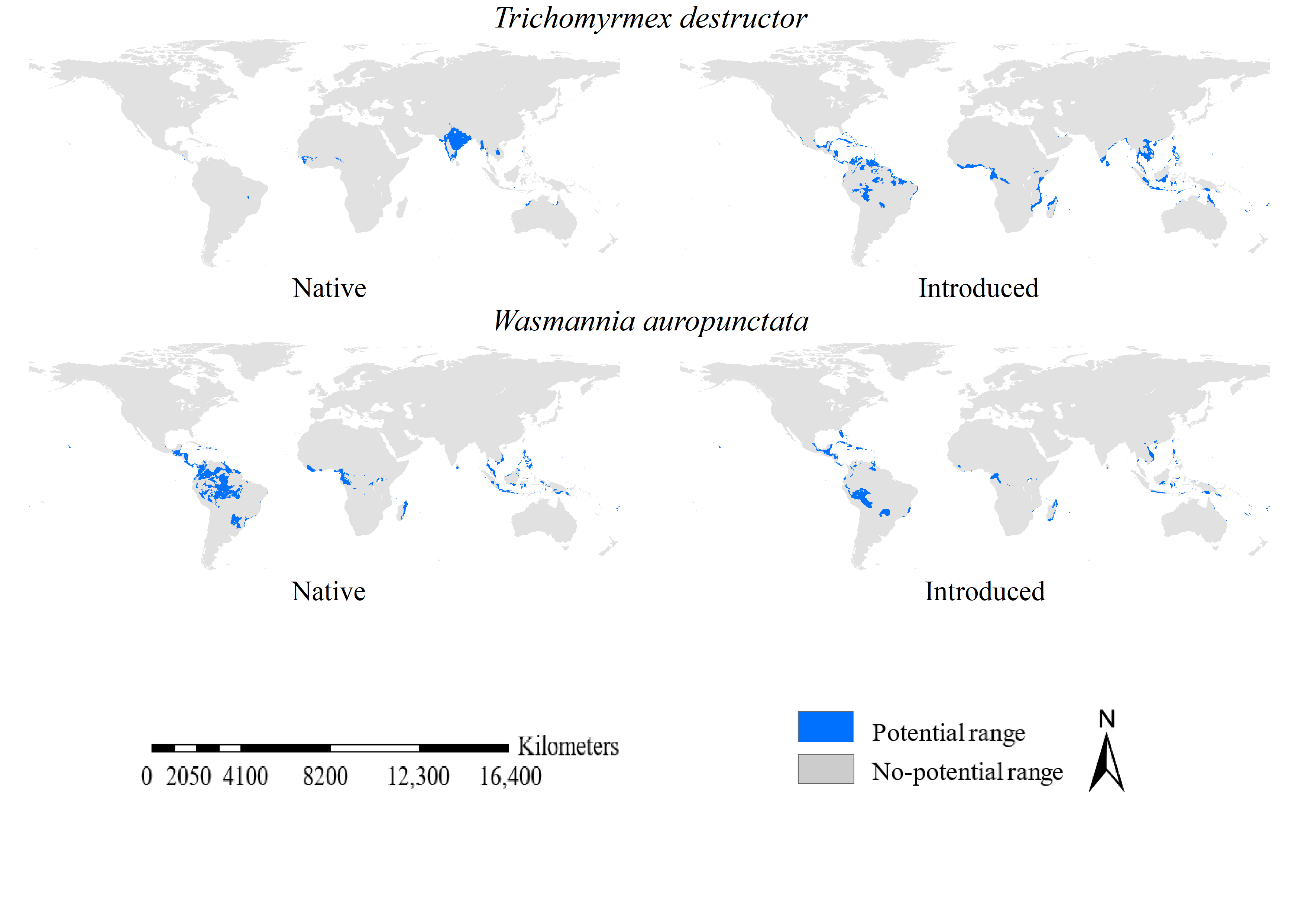


Expending Ranges of the major 18 ants


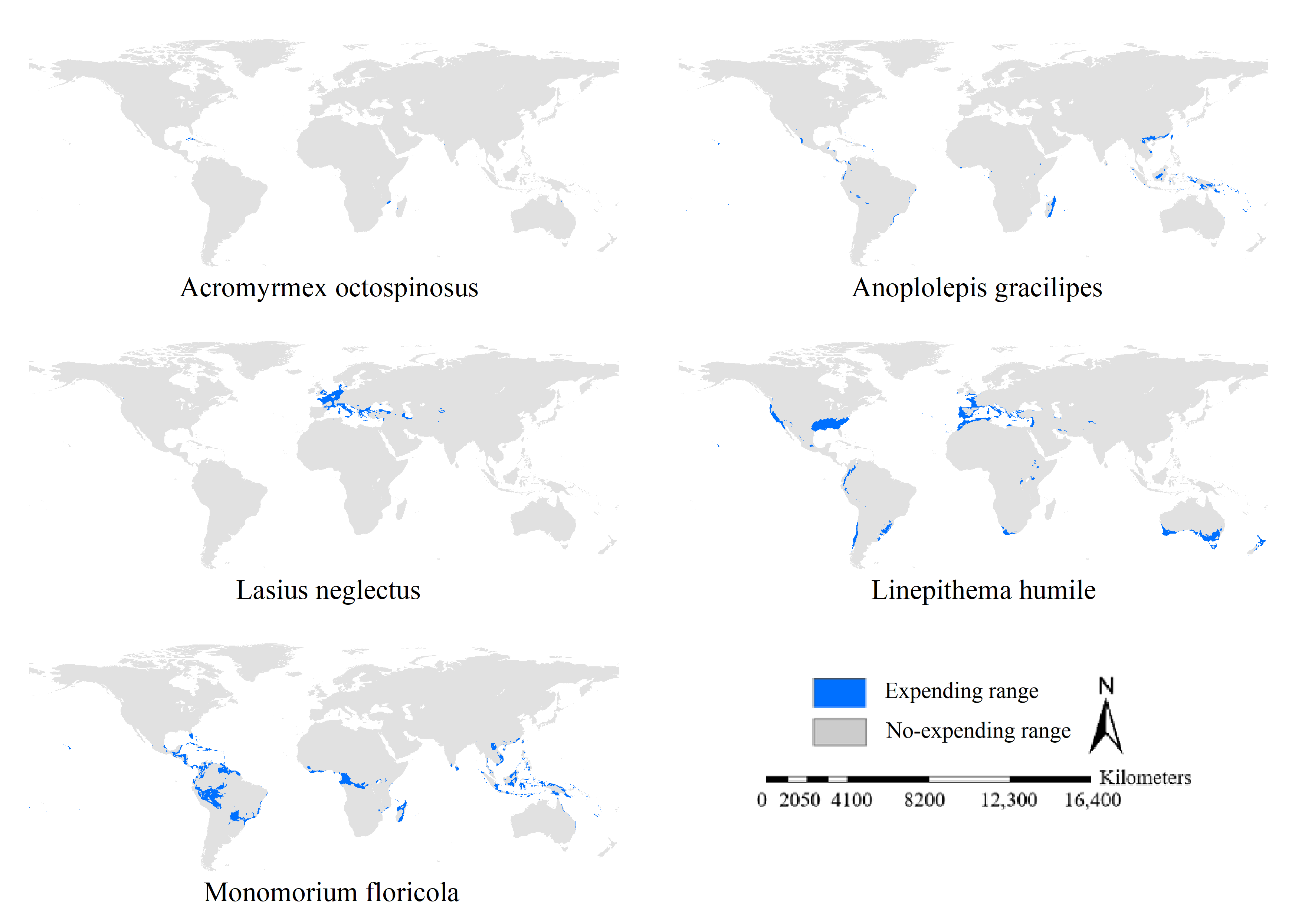


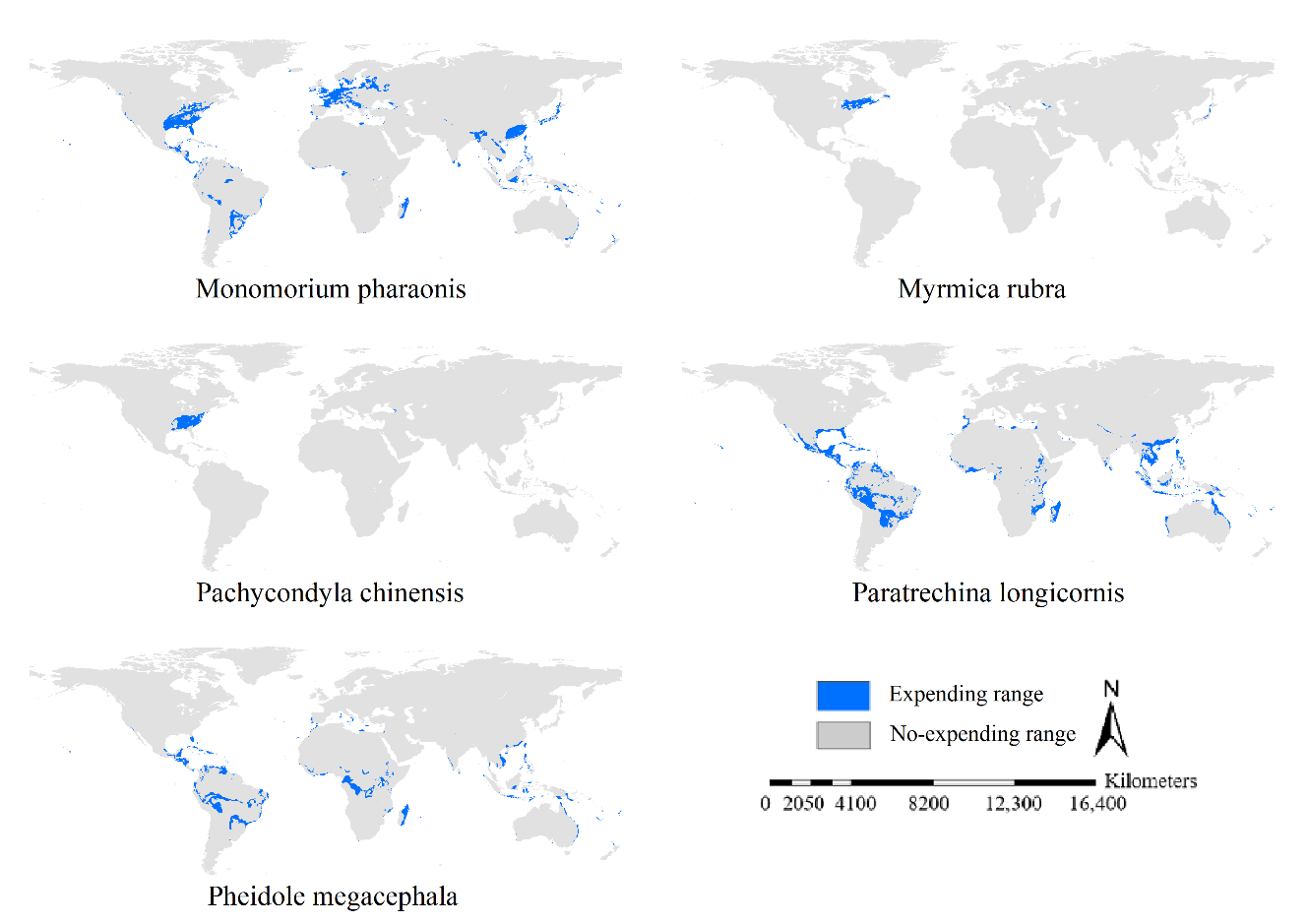


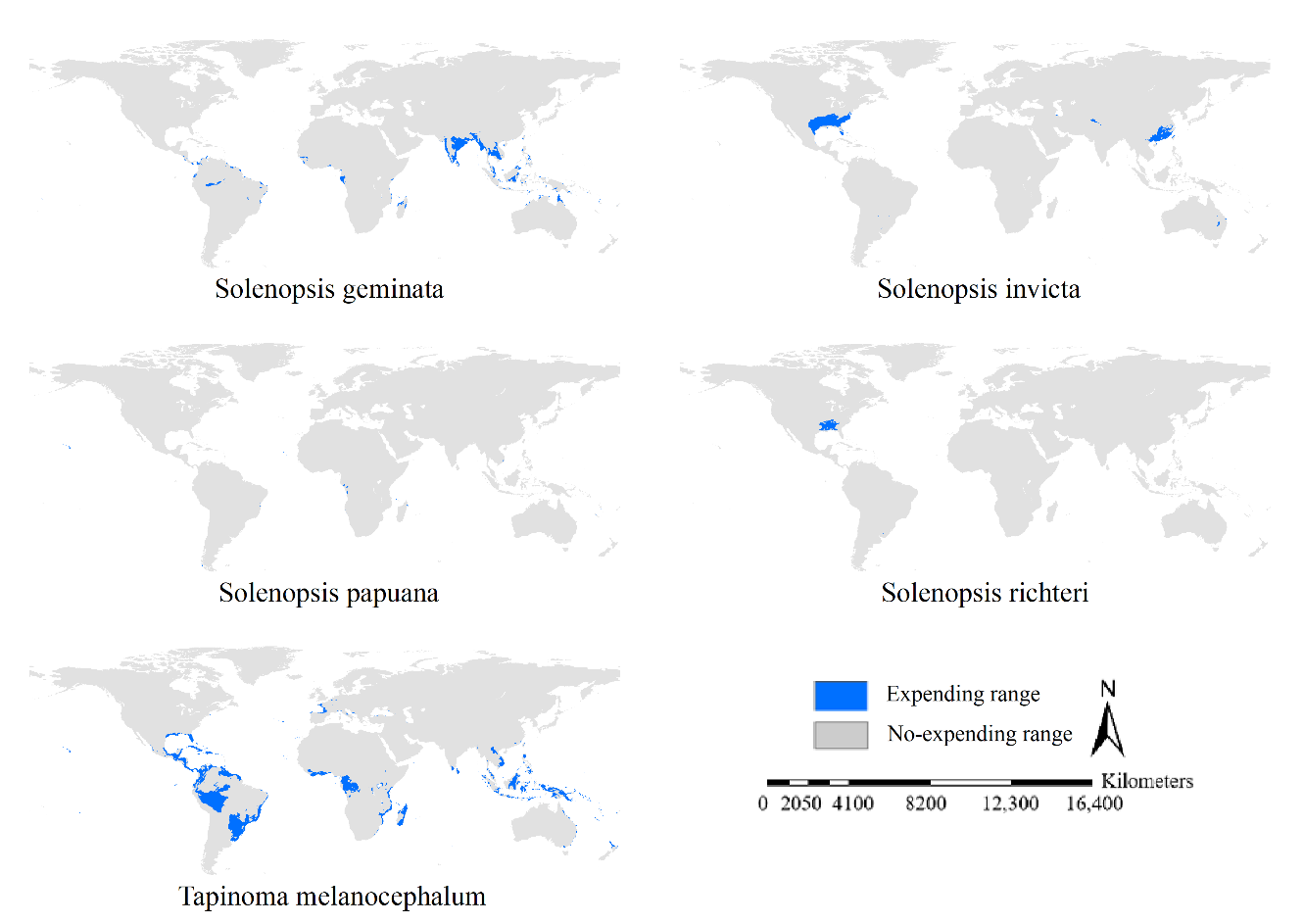


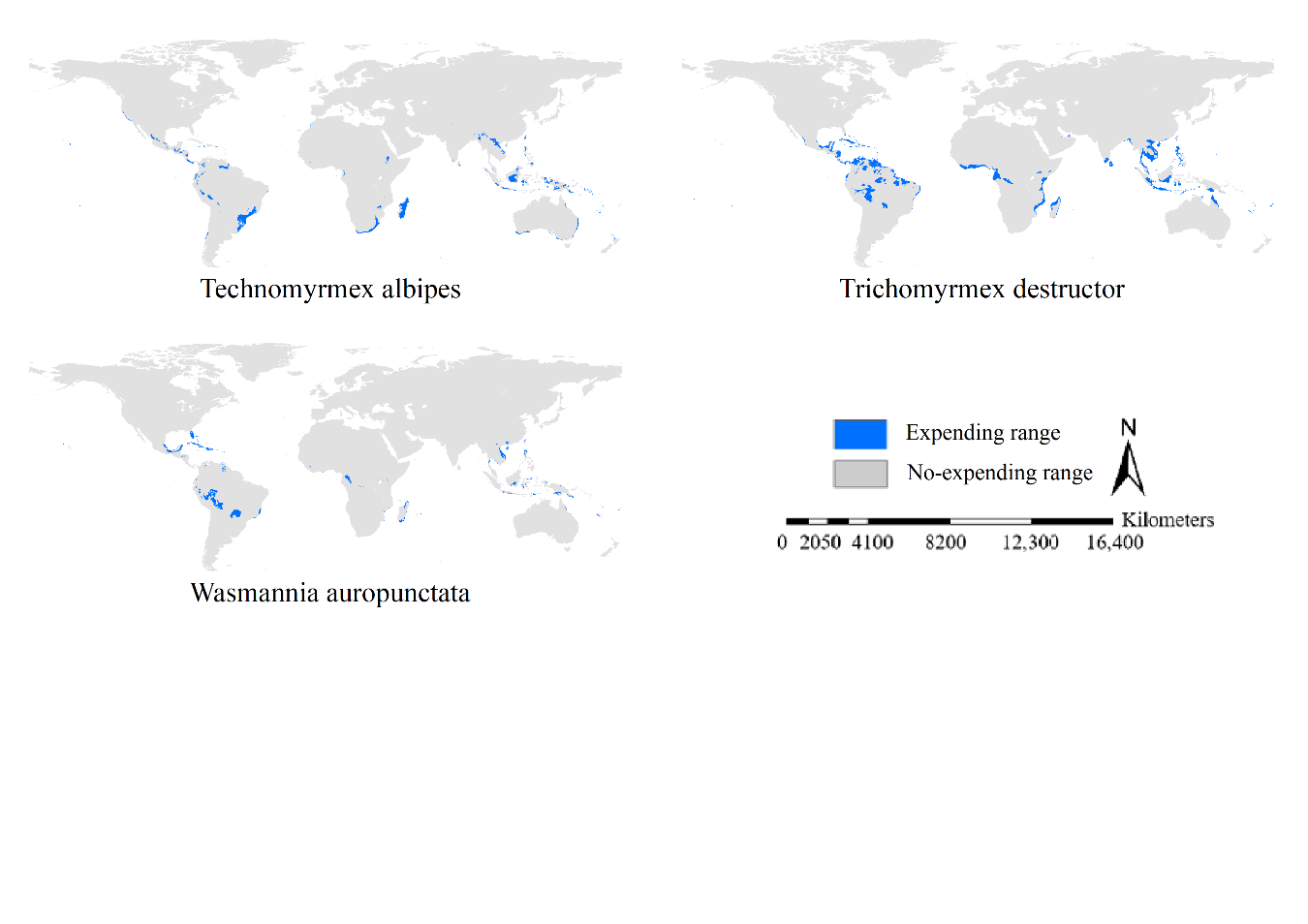

Supplement: Supplementary file 11 — Data S1. Potential ranges and expanding ranges. [file ECE3-15-e71754-s005.docx]
